# Endocannabinoid Signaling at Hypothalamic Steroidogenic Factor-1/Proopiomelanocortin Synapses Is Sex- and Diet-Sensitive

**DOI:** 10.3389/fnmol.2018.00214

**Published:** 2018-06-19

**Authors:** Carolina Fabelo, Jennifer Hernandez, Rachel Chang, Sakara Seng, Natalia Alicea, Sharon Tian, Kristie Conde, Edward J. Wagner

**Affiliations:** ^1^Department of Basic Medical Sciences, College of Osteopathic Medicine, Western University of Health Sciences, Pomona, CA, United States; ^2^Graduate College of Biomedical Sciences, Western University of Health Sciences, Pomona, CA, United States; ^3^College of Veterinary Medicine, Western University of Health Sciences, Pomona, CA, United States

**Keywords:** endocannabinoids, obesity, steroidogenic factor-1, sex differences, estradiol, proopiomelanocortin, testosterone, insulin

## Abstract

We tested the hypotheses that steroidogenic factor (SF)-1 neurons in the hypothalamic ventromedial nucleus (VMN) provide sexually disparate, endocannabinoid (EC)- and diet-sensitive glutamatergic input onto proopiomelanocortin (POMC) neurons. Electrophysiological recordings were performed in hypothalamic slices from intact and castrated guinea pigs, along with *in vitro* optogenetic experiments in intact male as well as cycling and ovariectomized female NR5A1-Cre mice. In slices from castrated male and female guinea pigs, depolarized-induced suppression of excitation (DSE) time-dependently reduced the amplitude of evoked excitatory postsynaptic currents (eEPSCs) in POMC neurons generated by electrically stimulating the dorsomedial VMN. Androgen stimulation rapidly enhanced this DSE, which was also found in insulin-resistant, high-fat diet (HFD)-fed males. By contrast, retrograde signaling at VMN/ARC POMC synapses was markedly attenuated in periovulatory females. HFD potentiated central cannabinoid-induced hyperphagia in both males and females, but exerted differential influences on cannabinoid-induced increases in energy expenditure. In NR5A1-Cre mice, the reduction in light-evoked EPSC amplitude caused by postsynaptic depolarization in cycling females was modest in comparison to that seen in intact males. Estradiol attenuated the DSE in light-evoked EPSC amplitude in slices from ovariectomized females. Moreover, the retrograde inhibition of transmission was further accentuated in HFD-fed males. Chemogenetic activation of SF-1 neurons suppressed appetite and increased energy expenditure in males, effects which were attenuated by HFD. Conversely, energy expenditure was increased in estradiol- but not vehicle-treated ovariectomized females. Together with our previous studies indicating that DSE in POMC neurons is EC-mediated, these findings indicate that VMN SF-1/ARC POMC synapses represent a sexually differentiated, EC- and diet-sensitive anorexigenic component within the hypothalamic energy balance circuitry.

## Introduction

Energy balance is regulated through interactions between the gastrointestinal tract (GI), brainstem and the hypothalamus. The hypothalamic energy balance circuity comprises the arcuate nucleus (ARC), ventromedial nucleus (VMN), lateral hypothalamus (LH), dorsomedial nucleus (DMN) and paraventricular nucleus (PVN) (Berthoud, 2012; Wilson and Enriori, 2015). The ARC is a critical mediator within this energy balance circuitry due to the precise location near the third ventricle in close contact to the median eminence (Belgardt et al., 2009). This allows the ARC to serve as a sensor of global energy homeostatic status of an organism (Belgardt et al., 2009; Berthoud, 2012; Wilson and Enriori, 2015).

Proopiomelanocortin (POMC)/cocaine amphetamine-regulated transcript (CART) neurons in the ARC are an critical anorexigenic component of the hypothalamic energy balance circuitry. The excitability of POMC neurons varies in direct proportion to the ambient glucose concentration (Ibrahim et al., 2003; Claret et al., 2007; Parton et al., 2007), and peripheral hormones such as insulin and leptin are known to influence the mRNA expression and excitability of POMC neurons (Belgardt et al., 2009). Insulin works through the mediobasal hypothalamus to increase lipogenesis and decrease lipolysis in white adipose tissue (Scherer et al., 2011). Leptin is secreted by adipocytes and circulating concentrations will increase with the accumulation of fat (Belgardt et al., 2009; Wilson and Enriori, 2015). Rodents and humans lacking leptin or a functional leptin receptor are immensely obese and hyperglycemic (Chua et al., 1996; Clément et al., 1998; Belgardt et al., 2009), while mice lacking the insulin receptor display a more mild obese phenotype (Brüning et al., 2000). Both leptin and insulin will depolarize POMC neurons via activation of transient receptor potential (TRP)C5 channels, which occurs through insulin and leptin receptor-mediated activation of phosphatidylinositol-3-kinase (PI3K) and leads to increased firing of POMC neurons (Cowley et al., 2003; Qiu et al., 2010, 2014). Leptin and insulin also modulate POMC neuronal firing by presynaptic action on neuropeptide Y (NPY)/agouti-related protein (AgRP) neurons, which will inhibit the release of gamma-amino butyric acid (GABA) from terminals impinging onto POMC neurons (Vong et al., 2011).

POMC neurons receive robust glutamatergic input from the dorsomedial portion of the VMN (Sternson et al., 2005; Krashes et al., 2014). A likely source of this glutamatergic input is from the population of steroidogenic factor (SF)-1 neurons emanating from that dorsomedial region of the VMN that project to the ARC and synapse onto POMC neurons (Lindberg et al., 2013; Cardinal et al., 2014). These neurons are exclusively found in the VMN (Majdic et al., 2002), express leptin, insulin and cannabinoid CB1 receptors, and when these cells are activated they suppress appetite and increase energy expenditure (Dhillon et al., 2006; Klöckener et al., 2011; Cardinal et al., 2014). These inputs are clearly part of what makes the VMN such an important anorexigenic component of the hypothalamic energy balance circuitry, and studies have shown that upon VMN lesion, hyperphagia and obesity occurs (Stricker, 1978).

The endocannabinoid system (ECS) exerts notable effects on energy homeostasis. Several studies have shown that a blockade of the CB1 receptor leads to a suppression of food intake, and central administration of CB1 receptor agonists increase food intake (Jamshidi and Taylor, 2001; Ravinet-Trillou et al., 2003; Verty et al., 2005). The two main endocannabinoids (ECs) are N-arachidonoylethanolamine (AEA) and 2-arachidonylglycerol (2-AG). Among the two, 2-AG is considered to be the most sensitive to changes induced by fasting and feeding in the hypothalamus (Di Marzo et al., 2001; Kirkham et al., 2002), although AEA but not 2-AG levels are elevated by fasting in the olfactory bulb (Soria-Gómez et al., 2014). ECs are known as retrograde messengers, and engage in a form of plasticity that occurs at glutamatergic and GABAergic synapses known as depolarization-induced suppression of excitation (DSE) or inhibition (DSI), respectively (Freund et al., 2003). For example, ECs retrogradely inhibit glutamatergic input onto POMC neurons in the ARC, and GABAergic transmission at melanin-concentrating hormone synapses in the LH (Jo et al., 2005; Borgquist et al., 2015a). EC levels are also influenced by anorexigenic/orexigenic factors such as leptin and ghrelin, respectively (Di Marzo et al., 2001; Kola et al., 2008). In addition, EC signaling is altered by obesity and type II diabetes, and may involve elevated EC tone (Di Marzo et al., 2001; Kim et al., 2013).

Many of the biological processes regulated by cannabinoids are sexually differentiated (Wagner, 2016). Our previous studies have shown that cannabinoid-induced hyperphagia and hypothermia is sexually disparate, with males being more sensitive than females (Diaz et al., 2009). Moreover, estradiol rapidly and markedly attenuates cannabinoid-induced hyperphagia in ovariectomized guinea pigs. It also decreases the magnitude and duration of the cannabinoid-induced hypothermia. These actions can be attributed, at least in part, to diminished cannabinoid-induced presynaptic inhibition of glutamatergic input onto POMC neurons (Kellert et al., 2009). The manner in which estradiol rapidly attenuates cannabinoid-induced presynaptic inhibition of excitatory input onto POMC neurons is due to activation of estrogen receptor subtype (ER)α and the G_q_-coupled membrane ER (mER) (Washburn et al., 2013). This attenuating effect of estradiol is due to signaling through two parallel pathways in POMC neurons: one involving protein kinase A and protein kinase C elicited following activation of the Gq-coupled mER, and another pathway involving PI3K and neuronal nitric oxide synthase (nNOS) elicited following activation of ERα. Moreover, estradiol down-regulates CB1 receptors in the hypothalamus (Washburn et al., 2013; Borgquist et al., 2015a; Mela et al., 2016).

By contrast, testosterone replacement in orchidectomized male guinea pigs increases energy intake, which is blocked by the CB1 receptor antagonist, AM251 (Borgquist et al., 2015b). Additionally, testosterone increases inhibitory GABAergic input onto POMC neurons as well as potentiates the EC-mediated retrograde inhibition of spontaneous excitatory postsynaptic current (EPSC) frequency and amplitude via DSE (Borgquist et al., 2015b). This potentiating effect of testosterone is blocked by the AMPK inhibitor compound C (Borgquist et al., 2015b), which indicates that AMPK plays an important role to the androgenic regulation of EC tone and energy homeostasis. Thus, the activational effects of gonadal hormones differentially modulate EC-mediated inhibition of excitatory input onto POMC neurons, which accounts, at least in part, for the prominent sex differences in the cannabinoid regulation of energy homeostasis. The question arises: from where does this EC-sensitive input originate? As mentioned above, there are several compelling reasons to think that SF-1 neurons in the dorsomedial VMN are a likely source. Therefore, we hypothesized that SF-1 neurons innervate ARC/POMC neurons and provide a source of sexually disparate, EC- and diet-sensitive glutamatergic input onto POMC neurons that serves as the basis for sex differences in the cannabinoid regulation for energy homeostasis.

## Materials and Methods

### Animal Models

Adult male and female Topeka guinea pigs (337–785 g; 35–103 days of age) were either purchased from Elm Hill Breeding Labs (Clemsford, MA, USA) or bred in-house. Intact female guinea pigs were checked daily through two consecutive estrous cycles to identify the period of genital swelling and vaginal opening. This was done to ensure that we performed the electrophysiological and behavioral testing during the periovulatory phase. Male and female NR5A1-Cre mice (18–43 g; 52–144 days of age) were generously provided by Drs. William Krause and Holly Ingraham at the University of California, San Francisco, CA, USA. Intact female NR5A1-Cre mice were checked the day of experimentation by vaginal lavage to evaluate cell cytology to determine the stage of the estrous cycle. Animals were housed under a 12:12 h light/dark cycle, with food and water available *ad libitum*. All procedures were approved by the Western University of Health Sciences’ IACUC in accordance with institutional guidelines based on NIH standards.

### Diet

Guinea pigs were subdivided and fed either a standard chow (Teklad Global Guinea Pig Diet, Madison, WI, USA), from which 12% of the calories were derived from fat, 31% from protein and 57% from carbohydrates or a “Westernized” high-fat diet (HFD; Newco Distributors Inc., CA, USA), from which 46% of the calories were derived from fat, 18% from protein and 36% from carbohydrates.

NR5A1-Cre mice were similarly subdivided and given continuous access to either a standard rodent chow (Teklad Rodent Diet, Teklad Diets, Madison, WI, USA) from which 18% of the calories were derived from fat, 24% from protein, and 58% from carbohydrates, or a high-fat diet (HFD; Research Diets, New Brunswick, NJ, USA) from which 45% of calories were derived from fat, 20% from protein and 35% from carbohydrates. All animals were kept on their respective diets for a minimum of 5 weeks prior to experimentation.

### Surgical Procedures

For some experiments, male guinea pigs were orchidectomized while they were under ketamine/xylazine (87.5 mg/kg and 12.5 mg/kg, respectively; s.c.) anesthesia maintained with 1.5%–2% isoflurane. For other experiments, NR5A1-Cre female mice were ovariectomized while they were under 2% isoflurane anesthesia.

The stereotaxic implantation of a guide cannula into the third ventricle of the guinea pig was performed as previously described (Borgquist et al., 2015a). Briefly, once anesthetized under ketamine/xylazine (87.5 mg/kg and 12.5 mg/kg, respectively; s.c.) maintained with 1.5%–2% isoflurane, an animal was secured in a stereotaxic frame (Stoelting, Wood Dale, IL, USA), and a midline incision was made through the scalp. A hole was then drilled in the skull, through which a 22-gauge guide cannula (Plastics One, Roanoke, VA, USA) was lowered 1 mm above the third ventricle at a 4° angle from the vertical plane using the following coordinates: AP −2.1 mm, ML ± 0.7 mm, DV −9.8 mm, tooth bar −5.5 mm. Three additional holes were drilled to insert anchor screws into the skull. The guide cannula was fastened in place with dental acrylic applied to the surgical field. Finally, a stylet was inserted into the guide cannula to keep the lumen patent. The animals were allowed to recover for 1–2 weeks prior to the start of experimentation.

To focally inject adeno-associated viral vector (AAV) constructs, NR5A1-Cre mice were anesthetized with 2% isoflurane and placed in a stereotaxic frame. An incision was made to expose the skull, and one or two holes were drilled on either side of the mid-sagittal suture so that an injection needle could be slowly lowered into the dorsomedial subdivision of the VMN using the following coordinates: AV −0.6 mm; ML, ± 0.3 mm; and DV, −5.6 mm. A unilateral injection of a Cre recombinase-dependent AAV vector containing cation channelrhodopsin-2 (ChR2; AAV1.EF1a.DIO.ChR2 (E123A).YFP.WPRE.jGH; 7.2 × 10^12^ genomic copies/mL; 300 nL total volume; University of Pennsylvania Vector Core; Addgene plasmid #35507), or a bilateral injection of a designer receptor exclusively activated by designer drugs (DREADD)-containing AAV vector (pAAV-EF1a-DIO-hM3D(Gq)-mCherry; 3.8 × 10^12^ genomic copies/mL; 300 nL total volume; University of North Carolina Vector Core; Addgene plasmid #50460) was given over 2 min. The injection needle remained in place for 10 min after infusion to allow for diffusion from the tip, and then slowly removed from the brain to reduce potential spread of the virus. Animals were used for experimentation 2–3 weeks after viral injection, and 1–2 weeks after gonadectomy.

### Drugs

All drugs were purchased from Tocris Bioscience/R&D Systems (Minneapolis, MN, USA) unless otherwise stated. For the behavioral experiments, estradiol benzoate (EB; Steraloids, Newport, RI, USA) was initially prepared as a 1 mg/mL stock solution in punctilious ethanol. A known quantity of this stock solution was added to a volume of sesame oil sufficient to produce a final concentration of 100 μg/mL, following evaporation of the ethanol. The cannabinoid receptor agonist (*R*)-(+)-[2,3-Dihydro-5-methyl-3-(4-morpholinylmethyl)pyrrolo[1,2,3-*de*]-1,4-benzoxazin-6-yl]- 1-naphthalenylmethanone mesylate (WIN 55,212-2) was dissolved in cremephor/ethanol/0.9% saline (CES; 1/1/18; v/v/v) at a concentration of 1.5 μg/μL, and delivered centrally in a total volume of 2 μL. Clozapine-N-oxide (CNO) was dissolved in filtered 0.9% saline to a final concentration of 0.3 mg/mL, and delivered systemically in a total volume of 1 mL/kg.

For the electrophysiological experiments, purified guinea pig insulin was purchased from Dr. Al Parlow (Harbor-UCLA Medical Center, Torrance, CA, USA) through the National Hormone and Peptide Program. It was dissolved in 0.01 HCl to a stock concentration of 20 μM, and further diluted with artificial cerebrospinal fluid (aCSF) to a working concentration of 20 nM. Tetrodotoxin (TTX; Alomone Labs, Jerusalem, Israel) was prepared as a 1 mM stock solution in NanoPure H_2_O, and diluted further with aCSF to the working concentration of 500 nM. The GABA_A_ receptor antagonist 6-imino-3-(4-methoxyphenyl)-1(6H)-pyridazinebutanoic acid hydrobromide (SR 95531) was dissolved in Ultrapure H_2_O to a stock concentration of 10 mM, and the stock solution was diluted further with artificial cerebrospinal fluid (aCSF) to the working concentration of 10 μM. The membrane impermeant source of testosterone 4-androsten-17 μ-ol-3-one-3 carboxymethyloxime bovine serum albumin (TBSA; Steraloids) was dissolved in DMSO to a stock concentration of 1 mM, and then diluted further with aCSF to a working concentration of 100 nM. The calcium/calmodulin inhibitor N-(10-aminodecyl)-5-chloro-1-naphthalenesulfonamide hydrochloride (A7) was dissolved in NanoPure H_2_O to a stock concentration of 3 mM, and further diluted into internal solution (see below), to a working concentration of 30 μM. 1,3,5(10)-Estratrien-3, 17β-diol (17β-estradiol; Steraloids) used for ovariectomized NR5A1-cre mice was dissolved in punctilious ethanol to a stock concentration of 1 mM, which was further diluted to a working concentration of 100 nM. The Akt activator 2-amino-6-chloro-α-cyano-3-(ethoxycarbonyl)-4H-1-benzopyran-4-acetic acid ethyl ester (SC79) was dissolved in NanoPure H_2_O to a stock concentration of 10 mM, and the stock concentration was diluted further with aCSF to a working concentration of 10 μM. The androgen receptor antagonist 2-methyl-N-(4-nitro-3-[trifluoromethyl]phenyl)propanamide (Flutamide) was dissolved in NanoPure H_2_O to a stock concentration of 10 mM, and further diluted with aCSF to a working concentration of 100 μM. The PI3K inhibitor 2-(4-morpholinyl)-8-(4-aminopheny)l-4H-1-benzopyran-4-one (PI 828) was dissolved in DMSO to a stock concentration of 10 mM, and further diluted with aCSF to a working concentration of 10 μM. CNO was dissolved in NanoPure H_2_O to a stock concentration of 5 mM, and further diluted with aCSF to its working concentration of 5 μM. All aliquots of the stock solutions were stocked at −20 degrees Celsius until needed for experimentation.

### Hypothalamic Slice Preparation

On the day of experimentation, the animal was briefly anesthetized with 32% isoflurane and rapidly decapitated. The brain was removed from the skull and the hypothalamic area was dissected. We then mounted the hypothalamic block on a cutting platform that was secured in a vibratome well filled with ice-cold, oxygenated (95% O_2_, 5% CO_2_) aCSF (for guinea pigs: NaCl 124, NaHCO_3_ 26, dextrose 10, HEPES 10, KCl 5, NaH_2_PO_4_ 2.6, MgSO_4_ 2, CaCl_2_ 1; for mice: sucrose 208, NaHCO_3_ 26, KCl, 2, NaH_2_ PO_4_ 1.25, dextrose 10, HEPES 10, MgSO_4_ 2, MgCl_2_ 1, CaCl_2_ 1; in mM). Four to five coronal slices (300 μm) through the rostrocaudal extent of the ARC were then cut. The slices were transferred to an auxiliary chamber containing room temperature oxygenated aCSF made like that described above for the guinea pig slice preparation and allowed to recover for a minimum of 1 h until the electrophysiological recording.

### Electrophysiology

Whole-cell patch clamp electrophysiological recordings from ARC neurons using biocytin-filled electrodes were performed in hypothalamic slices prepared from orchidectomized male guinea pigs, intact male guinea pigs, periovulatory female guinea pigs, gonadally intact male and cycling female NR5A1-Cre mice, and ovariectomized female NR5A1-Cre mice. During recordings, the slices were maintained in a chamber perfused with warmed (35 degrees Celsius), oxygenated aCSF in which we raised the CaCl_2_ concentration to 2 mM. Artificial CSF and all drugs (diluted with aCSF) were perfused via peristaltic pump at a rate of 1.5 mL/min. Patch electrodes were prepared from borosilicate glass (World Precision Instruments, Sarasota, FL, USA; 1.5 mm OD) pulled on a P-97 Flaming Brown puller (Sutter Instrument Co., Novato, CA, USA), and filled with an internal solution containing the following (in mM): cesium gluconate 128; NaCl 10; MgCl_2_ 1; EGTA 11; HEPES 10; ATP 1; GTP 0.25; 0.5% biocytin; adjusted to a pH of 7.3 with CsOH; osmolality: 286–320 mOsm. Electrode resistances varied from 3 MΩ to 8 MΩ.

For guinea pig experiments, whole-cell patch clamp recordings were performed using a Multiclamp 700A preamplifier (Axon Instruments, Foster City, CA, USA) that amplified potentials and passed current through the electrode. Membrane currents were recorded in voltage clamp with access resistances ranging from 8 MΩ to 20 MΩ. The signals underwent analog-digital conversion via a Digidata 1322A interface coupled to pClamp 10.5 software (Axon instruments). For the transgenic mouse experiments, recordings were made using an Olympus BX51 W1 fixed stage microscope outfitted with infrared differential interference contrast video imaging. A Multiclamp 700B preamplifier (Molecular Devices) amplified potentials and passed current through the electrode. Membrane currents underwent analog-digital conversion with a Digi-data 1550A interface (Molecular Devices) coupled to pClamp 10.5 software. The access resistance, resting membrane potential (RMP) and input resistance were monitored throughout the course of all recordings. If the access resistance deviated greater than 10% of the original value, the recording was ended. Low-pass filtering of the currents was conducted at a frequency of 2 kHz. The liquid junction potential was calculated to be −10 mV, and corrected for during data analysis using pClamp software. All recordings were performed under a holding potential of −75 mV. For all electrophysiological recordings, we generated an I/V relationship from a holding potential of −60 mV by delivering voltage commands at 10 mV increments (150 ms in duration) ranging from −50 mV to −130 mV. The expression of intrinsic currents such as the A-type K+ current and the hyperpolarization-activated mixed cation current (Ibrahim et al., 2003; Tang et al., 2005; Conde et al., 2016) provided the first indication that we were recording from POMC neurons.

To assess the postsynaptic actions of purified guinea pig insulin in POMC neurons from chow- and HFD-fed animals, recordings using the whole-cell configuration from holding potentials of −60 mV were performed. Insulin (20 nM) was perfused along with 500 nM TTX until a new steady-state holding current was established (6–8 min). Current-voltage (I/V) relationships were generated using a ramp protocol (75 mV/s) before and immediately following peptide application over a range of potentials extending from −100 mV to −10 mV.

To evoke EPSCs in the guinea pig model, we placed a bipolar tungsten stimulating electrode connected to a SUl5 stimulus isolation unit (Grass Telefactor, Warwick, RI, USA) into the dorsomedial portion of the VMN. Cells received a single stimulation of 18 V magnitude every 1–2 s for 500 μs delivered from a S88 Stimulus Generator (Grass Telefactor, W. Warwick, RI, USA). For the optogenetic experiments, recordings were performed in slices from NR5A1-Cre mice that were injected with a ChR2-containing viral vector into the VMN 2–3 weeks prior to experimentation. Once glutamatergic SF-1-expressing fibers (visualized with eYFP) impinging on ARC neurons were encountered, functional synaptic connectivity was ascertained by applying a photo-stimulus (25–100 ms pulses delivered every 2 s) from a light-emitting diode (LED) blue light source (470 nm) controlled by a variable 2A driver (ThorLabs, Newton, NJ, USA) that directly delivered the light path through the Olympus 40× water-immersion lens to generate a fast EPSC.

EC-mediated retrograde inhibition of excitatory input was assessed via DSE. To elicit the DSE, cells were given a 60-mV depolarization, which lasted 3 s in duration. These pulses were delivered every 60 s for up to 10–15 consecutive trials. After attaining 1–2 min of baseline eEPSC amplitude, we then began to execute the DSE protocol. For all experiments, the electrical stimulation and DSE protocol were executed in the presence of SR 95531(10 μM) to block GABA_A_ receptor-mediated synaptic input. We used TBSA (100 nM) to determine the potential for rapid membrane-delimited androgenic signaling, which was further evaluated using the androgen receptor antagonist flutamide (10 μM) in conjunction with TBSA (100 nM). A7 (30 μM) was applied via the internal solution in conjunction with bath application of TBSA (100 nm) to confirm the role of calcium/calmodulin in the androgenic enhancement of EC tone. To evaluate whether estradiol abrogates the DSE-changes in EC tone, and whether this occurs via PI3K, we bath administered 17β-estradiol (E_2_; 100 nM) to slices generated in ovariectomized NR5A1-Cre mice, or applied the PI3K inhibitor PI 828 (10 μM) to slices obtained from periovulatory, female guinea pigs. To assess the role of PI3K/Akt pathway in the change in EC tone caused by diet-induced obesity/insulin resistance, we bath applied the Akt inhibitor SC79 (10 μM) to slices obtained from chow- and HFD-fed male guinea pigs. Data were analyzed by examining the average post-stimulation amplitude acquired from at least three separate trials over 5 s bins that consisted of data out to 30 s after the DSE stimulus, and then normalizing that to the average EPSC amplitude collected during the baseline period.

### Immunohistochemistry

After recording, slices were then processed for immunohistochemistry using various phenotypic markers of ARC POMC neurons. Slices were fixed with 4% paraformaldehyde in Sorenson’s phosphate buffer (pH 7.4) for 120–180 min. The slices were then immersed overnight in 20% sucrose dissolved in Sorensen’s buffer, and frozen in Tissue-Tek embedding medium (Miles Inc., Elk-hart, IN, USA) the next day. Coronal sections (20 μm) were cut on a cryostat and mounted on chilled slides. These sections were then washed with 0.1 M sodium phosphate buffer (pH 7.4), and then processed with streptavidin-Alexa Flour (AF) 546 (Molecular Probes Inc., Eugene, OR, PA, USA) at a dilution of 1:600. After localizing the biocytin-filled neuron via fluorescence microscopy, the appropriate sections were processed further with polyclonal antibodies directed against α-melanocyte-stimulating hormone (α-MSH, Immunostar Inc., Hudson, WI, USA; 1:200 dilution), β-endorphin (Immunostar Inc.; 1:400 dilution), or cocaine and amphetamine-regulated transcript (CART; Phoenix Pharmaceuticals Inc., Burlingame, CA, USA; 1:200 dilution), and again evaluated using fluorescence immunohistochemistry. At the end of some of the behavioral experiments (see below), animals were anesthetized with 32% isofluorane and rapidly decapitated. Following brain removal, two to three coronal slices (1 mm in thickness) spanning the rostral-caudal extent of the mediobasal hypothalamus were prepared using a mouse brain matrix (Ted Pella Inc., Redding, CA, USA), submersed in Tissue-Tek embedding medium (Miles Inc.) and frozen in isopentane. Twenty micron-thick cryosections were then prepared for subsequent immunohistofluorescent determination of whether DREADD expression occurs in VMN SF-1 neurons using a polyclonal antibody directed against SF-1 and a monoclonal antibody against mCherry (Abcam, Cambridge, MA, USA; 1:300 and 1:500 dilutions, respectively).

### Feeding and Metabolic Studies

The feeding and metabolic studies were performed using a four-station Comprehensive Lab Animal Monitoring System (CLAMS; Columbus Instruments, Columbus, OH, USA) from which we monitored cumulative food intake, meal size, and several measures of energy expenditure (O_2_ consumption, CO_2_ production and respiratory exchange ratio (RER)) as previously described and validated (Farhang et al., 2010; Borgquist et al., 2015a; Conde et al., 2017). These studies were conducted under conditions in which food (standard chow or HFD) and water were available *ad libitum*. The animals were allowed to acclimate in their CLAMS chamber over 1–3 days. Each day they were weighed, handled and returned to their respective chambers. After the acclimation session, we initiated the 5-day monitoring phase during which the animals were weighed and injected each day and immediately placed back in their feeding chambers. For the guinea pig studies, intact males and periovulatory females were given either the CB1 receptor agonist WIN 55,212-2 (3 μg; I.3.V) or its CES vehicle (2 μL; I.3.V) each morning at 8:00 am. For the NR5A1-Cre mice, gonadally intact males and ovariactomized females were injected daily at 4:00 pm (2–3 h in advance of the nocturnal peak in energy consumption) with either CNO (0.3 mg/kg s.c.), or its 0.9% saline vehicle (1 mL/kg s.c.). Every other day, females were injected with EB (20 μg/kg; s.c.) or its sesame oil vehicle (1 mL/kg; s.c.). Monitoring took place continuously over 24 h. Cumulative food intake was taken as the total amount of food consumed at 1, 2 and 4 h after either CNO, EB or vehicle administration. Meal size is the amount of food eaten in a given hour divided by the number of meals in that same hour. The parameters of energy intake, meal pattern and energy expenditure were continuously written to a computer via an A/D converter.

### Statistical Analysis

Comparisons between two groups were made with either the Student’s *t*-test, or the Mann-Whitney U test. Comparisons made between more than two groups were performed using either the one-way, repeated measures multifactorial, or rank-transformed multifactorial analysis of variance (ANOVA) followed by the Least Significant Difference (LSD) test, or alternatively via the Kruskal-Wallis test followed by the median-notched box-and-whisker analysis. Differences were considered statistically significant if the alpha probability was 0.05 or less.

## Results

### Experiment #1: Does Diet Differentially Influence the Cannabinoid Regulation of Energy Homeostasis?

Previous studies have demonstrated that cannabinoid-induced changes in energy intake and core body temperature are sexually differentiated (Diaz et al., 2009), and elevated EC tone and signaling are implicated in diet-induced obesity/insulin resistance (Kim et al., 2013). We wanted to determine whether long-term exposure to a HFD modifies these sex differences. Our 5–8 week exposure paradigm produced frank sex differences in the development of central insulin resistance. This was characterized by the marked blunting of the amplitude of reversible, insulin-induced inward currents due to activation of TRPC channels in POMC neurons (Qiu et al., 2014, 2018) during recordings in slices from HFD-fed male but not periovulatory female guinea pigs (Supplementary Figure S1).

**Figure 1:**
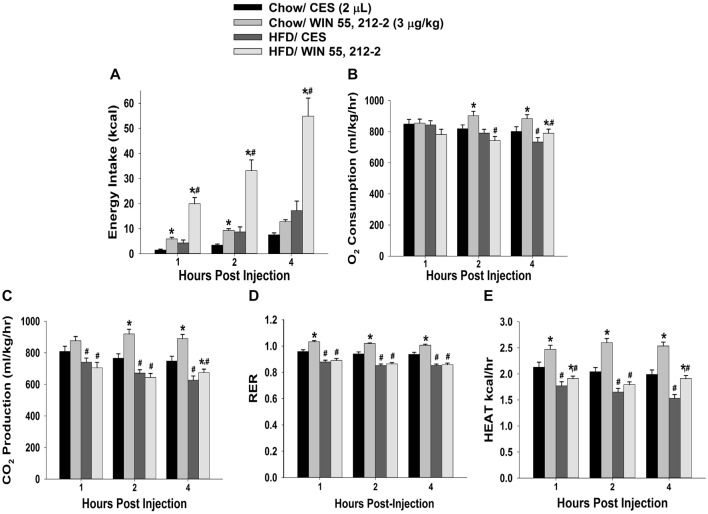
High-fat diet (HFD) potentiates cannabinoid-induced changes in energy intake and but not energy expenditure, in male guinea pigs. Bars represent means and vertical lines 1 SEM of the cumulative energy intake **(A)**, O_2_ consumption **(B)**, CO_2_ production **(C)**, respiratory exchange ratio (RER; **D)** and metabolic heat production **(E)**, which were measured 1, 2 and 4 h after administration of the cannabinoid receptor agonist WIN 55,212-2 (3 μg; I.3.V) or its cremephor/ethanol/0.9% saline (CES) vehicle (2 μL; I.3.V). *P < 0.05 relative to CES treated animals; multifactorial analysis of variance (ANOVA)/least significant difference (LSD); *n* = 5. ^#^*P* < 0.05 relative to chow-fed animals; repeated measures, multifactorial ANOVA/LSD; *n* = 5.

**Figure 2:**
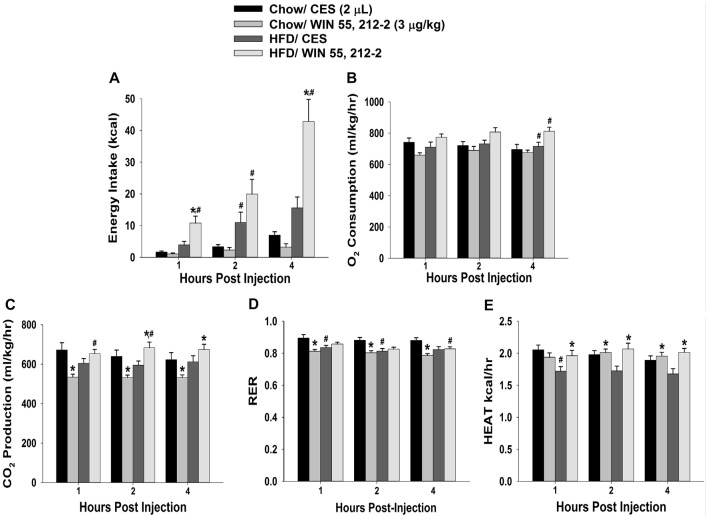
Activation of hypothalamic cannabinoid receptors increases energy intake and various measures of energy expenditure in HFD- but not chow-fed periovulatory female guinea pigs. Bars represent means and vertical lines 1 SEM of the cumulative energy intake **(A)**, O_2_ consumption **(B)**, CO_2_ production **(C)**, respiratory exchange ratio (RER; **D)** and metabolic heat production **(E)**, which were measured 1, 2 and 4 h after administration of WIN 55,212-2 (3 μg; I.3.V) or its CES vehicle (2 μL; I.3.V). *P < 0.05 relative to CES treated animals; multifactorial ANOVA/LSD; *n* = 5. ^#^*P* < 0.05 relative to chow-fed animals; repeated measures, multifactorial ANOVA/LSD; *n* = 5.

Despite these sex differences in the development of diet-induced insulin resistance, HFD clearly, yet disparately, impacted cannabinoid sensitivity in both males and females. We found significant main effects of sex and diet, and significant interactions between the two factors, on the ability of the cannabinoid receptor agonist WIN 55,212-2 (3 μg; I.3.V) to evoke changes in energy balance. WIN 55,212-2 increased cumulative energy intake in chow-fed males but not perivulatory females; however, the agonist-induced increase in intake was markedly accentuated in both HFD-fed male and periovulatory females (Figures 1A, 2A). The HFD-induced potentiation of the hyperphagia caused by WIN 55,212-2 is associated with heightened increases in meal size in both males and periovulatory females (not shown). On the other hand, WIN 55,212-2 increased O_2_ consumption in chow-fed males, an effect that was antagonized by the HFD, whereas the agonist was without effect on O_2_ consumption in either HFD- or chow-fed periovulatory females (Figures 1B, 2B). WIN 55,212-2 also increased CO_2_ production in chow-fed males. HFD *per se* decreased CO_2_ production, and prevented the agonist-induced increase seen in the chow-fed animals. By contrast, WIN 55,212-2 decreased CO_2_ production in chow-fed periovulatory females, an effect that was entirely reversed by the HFD (Figures 1C, 2C). In addition, WIN 55,212-2 elevated RER in chow-fed males. HFD *per se* lowered the RER, and blocked the increase caused by the agonist. Conversely, WIN 55,212-2 reduced the RER in chow-fed periovulatory females. HFD *per se* also decreased the RER, and prevented any further decrease caused by the agonist (Figures 1D, 2D). Lastly, WIN 55,212-2 increased metabolic heat production in chow-fed males. HFD *per se* lowered metabolic heat production, and markedly attenuated the agonist-induced increase. In periovulatory females, the HFD *per se* also reduced metabolic heat production; however, in contrast to the male, WIN 55,212-2 increased metabolic heat production in both HFD- and chow-fed animals (Figures 1E, 2E). Thus, diet disparately modulates sex differences in the cannabinoid regulation of energy intake and expenditure.

### Experiment #2: Is There a Sex Difference in Endocannabinoid Signaling at VMN/ARC POMC Synapses, and Does it Involve Rapid Enhancement Through Membrane-Initiated Androgen Receptor Signaling?

**Figure 3:**
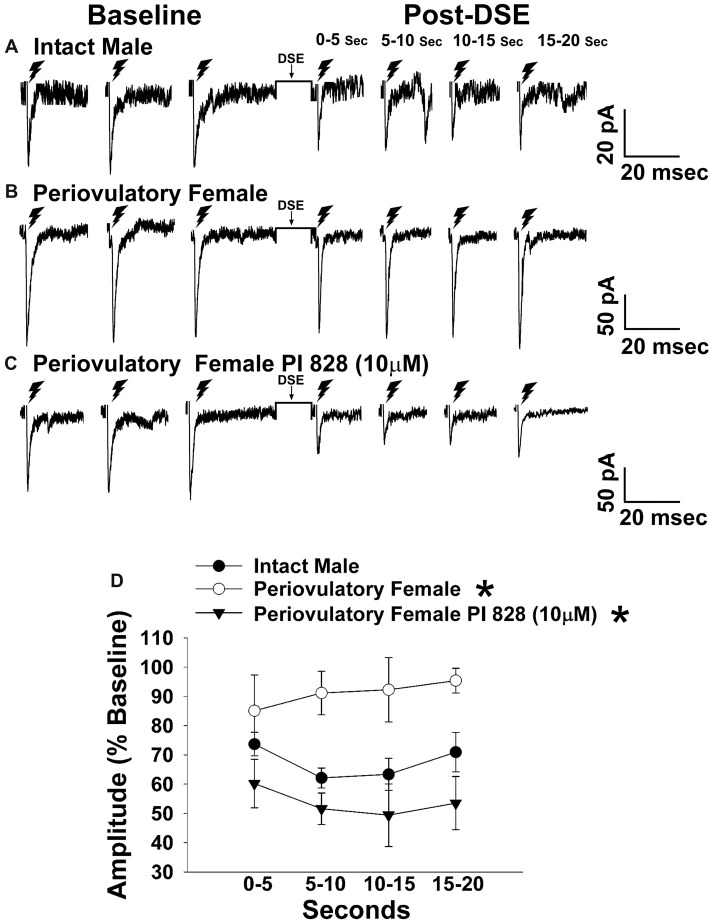
The postsynaptic depolarization-induced reduction in excitatory input onto proopiomelanocortin (POMC) neurons generated by stimulation of the dorsomedial ventromedial nucleus (VMN) is larger in males than in periovulatory females, which is reversed by the phosphatidylinositol-3-kinase (PI3K) inhibitor PI 828. Membrane current traces illustrating the baseline evoked excitatory postsynaptic current(eEPSC) amplitude, and the time-dependent reduction in amplitude at various time points after the depolarized-induced suppression of excitation (DSE) stimulus, in arcuate nucleus (ARC) POMC neurons are shown for the intact male (**A**; *n* = 9) periovulatory female (**B**; *n* = 6) and a slice from a periovulatory female pretreated with PI 828 (10 μM; **(C)** *n* = 4). The composite line graph **(D)** further illustrates the PI3K-dependent sex differences in the retrograde inhibition of excitatory input from the dorsomedial VMN onto ARC POMC neurons under baseline conditions. Lines represent means and lines 1 SEM. *P < 0.05; rank-transformed multifactorial ANOVA/LSD; *n* = 4–9.

The sex difference in the cannabinoid regulation of energy balance can be attributed, at least in part, to disparites in the presynaptic inhibition of glutamatergic input onto POMC neurons (Wagner, 2016). Evidence suggests that the VMN provides a prominent excitatory input that impinges on POMC neurons (Sternson et al., 2005; Krashes et al., 2014). Therefore, to verify the anatomical origin of this cannabinoid-sensitive glutamatergic input, we attempted to evoke EPSCs generated by electrical stimulation of the dorsomedial VMN. We recorded from a total of 132 guinea pig ARC POMC neurons. Stimulation of the dorsomedial VMN in slices from chow-fed gonadally intact males and periovulatory females elicited robust eEPSCs. The amplitude of the eEPSCs observed in POMC neurons generated by electrical stimulation of the dorsomedial VMN exhibited a reduction caused by postsynaptic depolarization in a sex-dependent manner. Thus, the extent of the postsynaptic depolarization-induced reduction in eEPSC amplitude was greater in males than in periovulatory females, an effect that was reversed in recordings from periovulatory females that were pretreated with the PI3K inhibitor PI 828 (Figure 3). These data suggest that this glutamatergic input impinging onto POMC neurons in the ARC is significantly attenuated by EC signaling in males compared to females, and that PI3K signaling contributes to this disparity. Due to this apparent sexual disparity, we decided to turn our attention to the androgenic influence on EC signaling within VMN/ARC POMC synapses.

**Figure 4:**
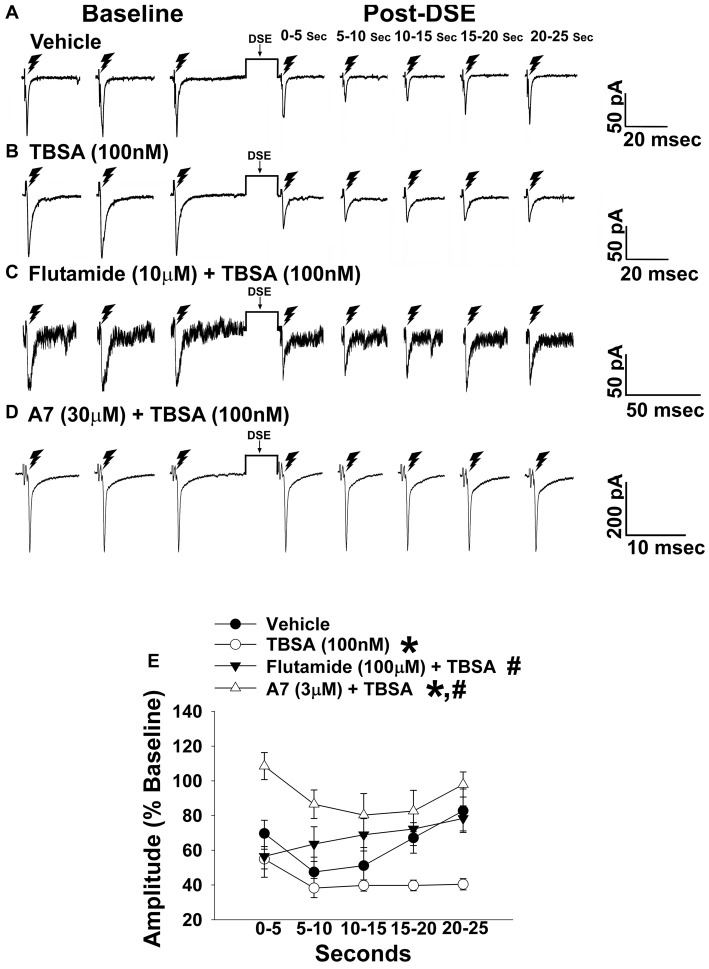
The membrane-impermeant form of testosterone rapidly intensifies endocannabinoid (EC)-mediated reduction in eEPSC amplitude, which is blocked by a classical androgen receptor antagonist, as well as via inhibition of calcium/calmodulin-dependent protein kinase. Current membrane traces showing the decrease in eEPSC amplitude caused by postsynaptic depolarization seen in slices treated with either vehicle (**A**; *n* = 6) or testosterone 4-androsten-17 μ;-ol-3-one-3 carboxymethyloxime bovine serum albumin (TBSA; 100 nM; **B**; *n* = 8). Bath application of flutamide (10 μM) attenuated the rapid potentiating effects of TBSA (100 nM; **C**; *n* = 5) on the reduction in eEPSC amplitude, as did the calcium/calmodulin antagonist A7 administered via the internal solution (30 μM; **D**; *n* = 5). The composite line graph **(E)** shows the enhanced retrograde inhibition caused by TBSA. *P < 0.05 relative to vehicle treated slices; rank-transformed multifactorial ANOVA/LSD. ^#^*P* < 0.05 relative to slices treated with TBSA alone; rank-transformed multifactorial ANOVA/LSD.

In recordings from males that were orchidectomized in order to remove any confounding influences caused by endogenously produced testosterone, significant main effects of steroid and time were encountered with bath application of the membrane impermeant TBSA (100 nM), which prolonged the extent of the reduction caused by postsynaptic depolarization (Figure 4). These data indicate that androgens are involved in increasing EC tone. Due to the rapid time scale over which the TBSA augmented the postsynaptic depolarization-induced reduction in eEPSC amplitude, the possibility exists that this is due to a membrane bound androgen receptor. To further confirm this, we bath applied the classical androgen receptor antagonist flutamide (100 nM; Figure 4) along with TBSA. A significant main effect of treatment was found, such that the rapid androgenic augmentation of the reduction in eEPSC amplitude caused by postsynaptic depolarization was blocked with flutamide. Similarly, the calcium/calmodulin antagonist A7 (30 μM; Figure 4) abrogated the TBSA-induced enhancement of the decrease in eEPSC amplitude caused by DSE. These data provide further evidence that increases in intracellular Ca^2+^, in conjunction with the activation of calcium/calmodulin-dependent protein kinase account, at least in part, for the rapid androgenic potentiation of EC-mediated retrograde inhibition. Given this androgenic enhancement of EC tone onto VMN/ARC POMC synapses, which can also be heightened under metabolic conditions such as diet-induced obesity/insulin resistance (Kim et al., 2013), it was of interest to see how diet would influence glutamatergic neurotransmission within this circuit.

### Experiment #3: Does Diet Differentially Alter Endocannabinoid Signaling at VMN/ARC POMC Synapses via Alterations in PI3K/Akt Signaling?

**Figure 5:**
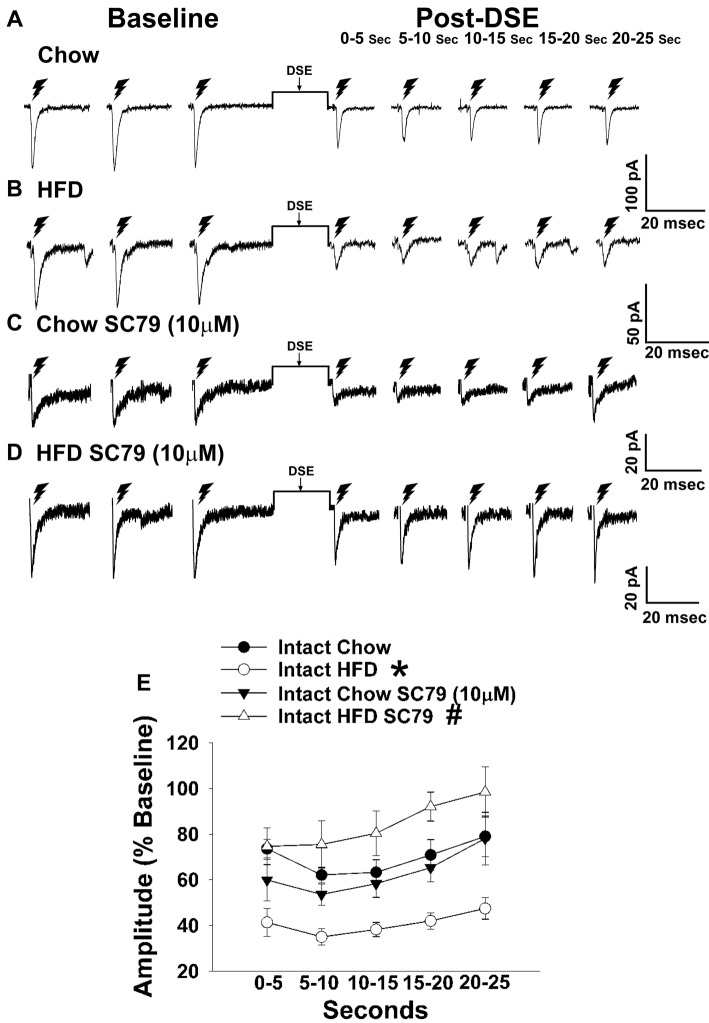
HFD intensifies the reduction in eEPSC amplitude caused by postsynaptic depolarization, which is abrogated by Akt activation. The reduction in eEPSC amplitude caused by the DSE seen in chow-fed animals (**A**; *n* = 9) is further amplified in HFD-fed animals (**B**; *n* = 5). The reduction in eEPSC amplitude caused by postsynaptic depolarization seen during recordings in slices taken from chow-fed animals is unaffected by pretreatment with the Akt activator SC79 (10 μM; **C**; *n* = 6). However, the heightened retrograde signaling observed in slices from HFD-fed animals **(B)** was completely abolished with SC79 (**D**; *n* = 5). The composite line graph further illustrates the heightened postsynaptic depolarization-induced reduction in eEPSC amplitude caused by the HFD, and the impact of reduced PI3K/Akt signaling on the augmented EC tone **(E)**. Lines represent means and vertical lines 1 SEM. *P < 0.05 relative to chow-fed animals; rank-transformed multifactorial ANOVA/LSD. ^#^*P* < 0.05 relative to vehicle-treated slices; rank-transformed multifactorial ANOVA/LSD.

Long-term exposure to a “Westernized” HFD causes sexually differentiated insulin resistance that, among other things, is associated with a reduced insulin receptor-mediated activation of TRPC5 channels in POMC neurons from male but not female guinea pigs (Supplementary Figure S1). In addition, the data shown in Figures 1, 2 above clearly indicate that diet can profoundly impact the cannabinoid-induced changes in energy intake and expenditure seen in males and females. This suggests that diet-induced insulin resistance could manifest as a dysregulated “sensing” of energy balance—brought on by enhanced cannabinoid sensitivity that creates a state of negative energy balance within the homeostatic energy balance circuitry. We then attempted to resolve these differential dietary influences down to the level of the hypothalamic energy balance circuitry. To test the hypothesis that long-term HFD exposure augments EC signaling within VMN/ARC POMC synapses, we generated eEPSCs via electrical stimulation of the dorsomedial VMN in slices from gonadally intact, chow- or HFD-fed male and periovulatory female guinea pigs. Males fed a HFD exhibited a significant eEPSC amplitude reduction following the DSE as compared to those fed a standard-chow diet (Figures 5A,B). Conversely, only a very small percentage (20%) of POMC neurons from HFD-fed periovulatory females displayed any eEPSCs in response to stimulation of the dorsomedial VMN (Supplementary Table S1); effectively precluding any assessment of EC signaling at VMN/ARC POMC synapses in these animals. These data show that a prolonged exposure to a HFD leads to an increase in EC tone and retrograde inhibition that decreases glutamatergic input onto POMC neurons in males; however, in periovulatory females this may involve a dramatic reduction in the number of excitatory inputs emanating from the dorsomedial VMN.

Long-term exposure to a HFD is associated with reduced PI3K/Akt signaling in the ARC of gonadally intact male but not periovulatory female guinea pigs (Qiu et al., 2018). To determine if this reduction in PI3K/Akt signaling contributes to the HFD-induced enhancement of EC tone at VMN/ARC POMC synapses, we then performed recordings in slices from chow- and HFD-fed intact males pre-treated with the Akt activator SC79 (10 μM) or its vehicle. A significant main effect of SC79, as well as a significant interaction between SC79 and diet, were observed. Hence, the postsynaptic depolarization-induced reduction in eEPSC amplitude seen in recordings in slices from chow-fed animals pre-treated with SC79 is retained (Figure 5C). However, in animals fed a HFD this effect is completely abrogated (Figure 5D). The composite line graph seen in Figure 5E further illustrates the fact that retrograde, EC-mediated decreases in eEPSC amplitude (Conde et al., 2017) were significantly greater in HFD-fed animals compared to chow-fed controls at each time bin, an effect that was completely abolished in the presence of SC79. These data indicate that the HFD-induced enhancement of retrograde, EC-mediated signaling at VMN/ARC POMC synapses is facilitated by the reduction in Akt activation within the mediobasal hypothalamus.

### Experiment #4: Does Photostimulation of VMN SF-1 Neurons Elicit a Physiologically Relevant, Sexually Differentiated and EC-Sensitive Source of Glutamatergic Input Onto ARC POMC Neurons?

To this point, we have shown that electrical stimulation of the dorsomedial VMN during electrophysiological recordings from slices obtained from male and female guinea pigs creates a robust eEPSC response. The amplitude is then reduced in a time-dependent manner through retrograde, EC-mediated inhibition of excitatory input onto POMC neurons.

**Figure 6:**
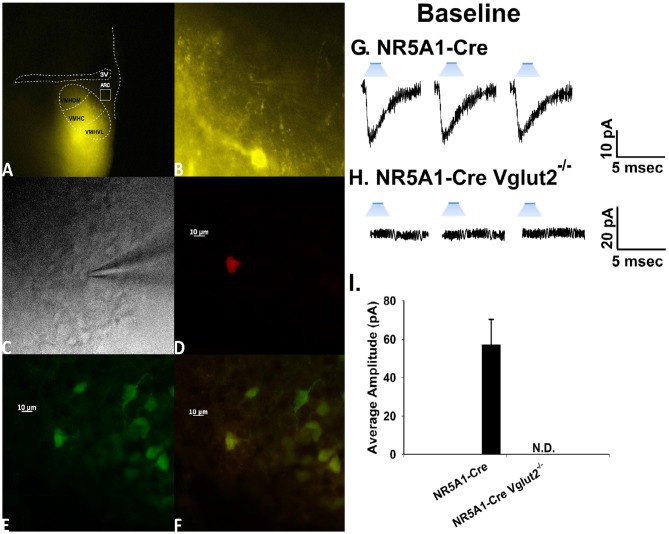
Photostimulation elicits glutamatergic EPSCs at VMN steroidogenic factor (SF-1)/ARC POMC synapses. **(A)** ChR2 labeling in the VMN of a male NR5A1-Cre mouse visualized at ×4 with enhanced yellow fluorescent protein (eYFP) 2 weeks after injection with a ChR2-containing virus. Of note is the gradient extending from the dorsomedial VMN (VMHDM) to the ventrolateral VMN (VMHVL) that is characteristic of the distribution of VMN SF-1 neurons. **(B)** Magnified view of the outlined approximate recorded area in the ARC from **(A)** showing labeling of ChR2-containing fibers in the ARC visualized with eYFP. **(C)** An infrared, differential interference contrast image taken of an ARC neuron in close proximity to the eYFP-labeled fibers seen in **(B)**. **(D)** Biocytin labeling of the cell in **(A)** visualized with streptavidin/Alexa Fluor 546. **(E)** An antibody directed against cocaine- and amphetamine-regulated transcript (CART), a phenotypic marker of POMC neurons, immunolabels the cell in **(C)** as visualized with Alexa Fluor 488. **(F)** A composite overlay of the biocytin/CART labeling seen in the cell in **(A)**. Unless otherwise indicated, all photomicrographs were taken at ×40.A 25-ms stimulus of blue light subsequently delivered to the cell in **(C–F)** elicited robust light-evoked EPSCs (leEPSCs; **G)** that were indiscernible in recordings from NR5A1-cre^Vglut2−/–^ animals **(H)**. This is further illustrated in the composite bar graph in **(I)** Bars represent means and lines 1 SEM of the EPSC amplitude measured in slices from NR5A1-Cre (*n* = 22) and NR5A1-Cre^Vglut2−/–^ (*n* = 3) animals.

To confirm the excitatory transmission evoked by electrical stimulation of the dorsomedial portion of the VMN is indeed coming from SF-1 neurons, we utilized the transgenic NR5A1-Cre mouse model. We recorded from a total of 137 ARC POMC neurons in these mice, an example of which is represented in Figure 6. These animals express cre-recombinase under the control of the NR5A1 promoter, which is the gene that encodes for the SF-1 transcription factor. We injected the VMN with an AAV construct containing ChR2 tagged with a YFP reporter to activate the SF-1 neurons through photstimulation. Two to three weeks later, we performed visualized, whole-cell patch clamp recordings in ARC neurons subsequently identifed as POMC neurons via immunohistofluorescence (Figures 6A,F). Upon optogenetic photostimulation, robust light-evoked EPSCs (leEPSCs) are seen (Figures 6G,I). By contrast, recordings from NR5A1-Cre mice in which the glutamate transporter Vglut2 is selectively knocked out in SF-1 neurons failed to exhibit any leEPSCs upon photostimulation (Figures 6H,I). These results provide a clear indication that glutamatergic input onto POMC neurons is indeed emanating from SF-1-containing neurons in the dorsomedial portion of the VMN. As such, these VMN SF-1/ARC POMC synapses constitute a fundamental component of the hypothalamic energy balance circuitry.

**Figure 7:**
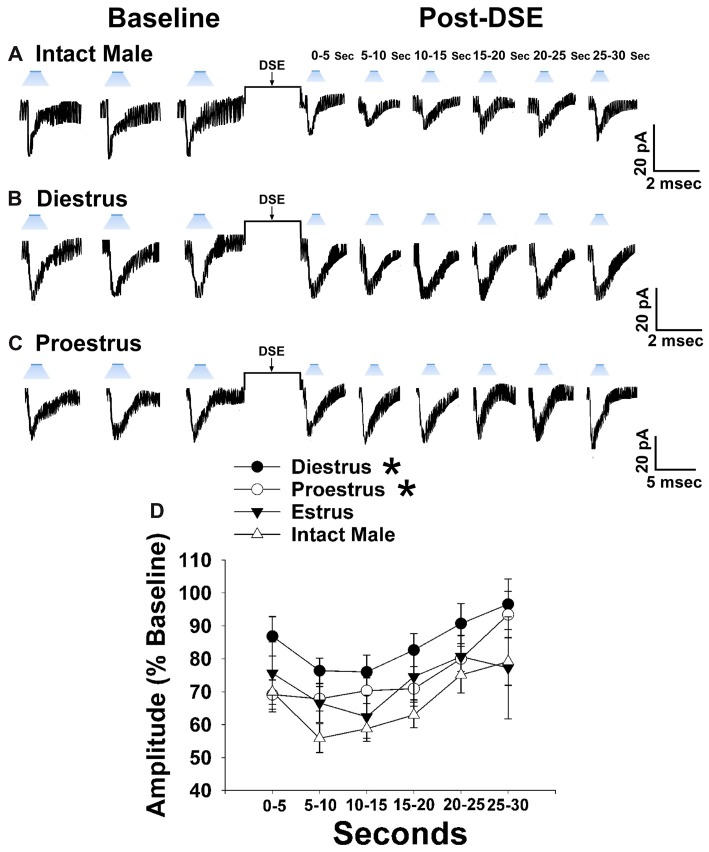
Sex differences in the retrograde inhibition of glutamatergic input at VMN SF-1/ARC POMC synapses in NR5A1-Cre mice. The representative traces of membrane current show that the reduction in leEPSC amplitude caused by postsynaptic depolarization is comparatively larger in gonadally intact males (**A**; *n* = 22) than in diestrus (**B**; *n* = 19) and proestrus (**C**; *n* = 21) females. This can also be seen from the composite line graph **(D)**. Lines represent means and lines 1 SEM. *P < 0.05; rank-transformed multifactorial ANOVA/LSD.

Now that we have clearly demonstrated that VMN SF-1 neurons provide a robust source of glutamatergic input onto POMC neurons, we endeavored to explore whether this input is EC-sensitive and, if so, whether the associated retrograde inhibition is sexually differentiated. Figure 7 shows representative leEPSCs evoked under basal conditions, and at various time points after the DSE stimulus, during recordings in slices taken from NR5A1-Cre females across the estrous cycle. Recordings during metestrus revealed a significantly lower percentage of cells exhibiting leEPSCs under basal conditions (see Supplementary Table S2). Rank-transformed multifactorial ANOVA revealed significant main effects of sex/cycle and time. The degree of the reduction in leEPSC amplitude caused by postsynaptic depolarization seen in recordings from diestrus (Figure 7B) and proestrus (Figure 7C) female NR5A1-Cre slices is modest at best. This is especially so when one considers the postsynaptic depolarization-induced reduction in leEPSC amplitude seen during recordings from gonadally intact NR5A1-Cre males (Figure 7A). Indeed, the decrease in leEPSC amplitude is significantly greater in intact males than in diestrus or proestrus females (Figure 7D).

### Experiment #5: Does 17β-Estradiol Attenuate Retrograde, EC-Mediated Inhibition of Glutamatergic Input at VMN SF-1/ARC POMC Synapses?

**Figure 8:**
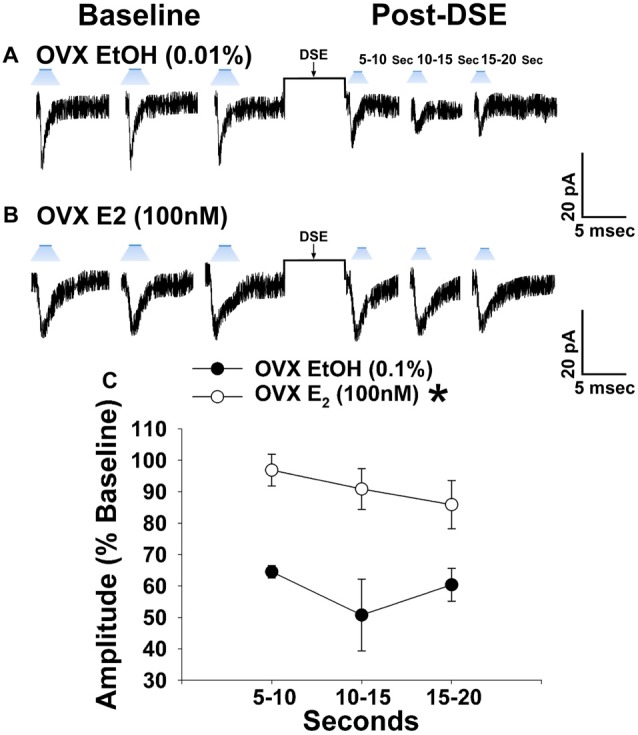
E_2_ significantly attenuates retrograde EC inhibition of leEPSC amplitude in POMC neurons from ovariectomized NR5A1-cre mice. LeEPSC amplitude is reduced post-DSE in recordings from EtOH-treated slices (0.01%; **A**; *n* = 5). However when other slices are treated with E_2_ (100 nM; *n* = 7), the degree of reduction is significantly reduced **(B)**. These points are further illustrates by the data shown in the line graph in **(C)** Bars represent means and vertical lines 1 SEM. *P < 0.05; rank-transformed multifactorial ANOVA/LSD.

Due to the comparatively modest reduction in leEPSC amplitude caused by the DSE stimulus across the various stages of the estrous cycle, we ovariectomized female NR5A1-Cre mice to determine putative activational effects of ovarian hormones in an environment devoid of any endogenous production. 17β-Estradiol (E_2_) has long been known to uncouple G_i/o_-linked receptors from their effector systems (Lagrange et al., 1994, 1996; Qiu et al., 2003). Therefore, we tested the hypothesis that E_2_ would attenuate EC signaling at VMN SF-1/ARC POMC synapses. Slices treated with E_2_ exhibited a significantly muted decrease in leEPSC amplitude following DSE as compared to those treated with its EtOH vehicle (Figure 8). These data indicate that estradiol exerts important activational effects that limit the retrograde inhibition of glutamatergic input onto POMC neurons. This would account, in part, for the sex differences in EC signaling at VMN SF-1/ARC POMC synapses.

### Experiment #6: Is EC-Mediated Retrograde Inhibition Occurring at VMN SF-1/ARC POMC Synapses Disparately Enhanced by Diet-Induced Obesity in Male and Female NR5A1-Cre Mice?

**Figure 9:**
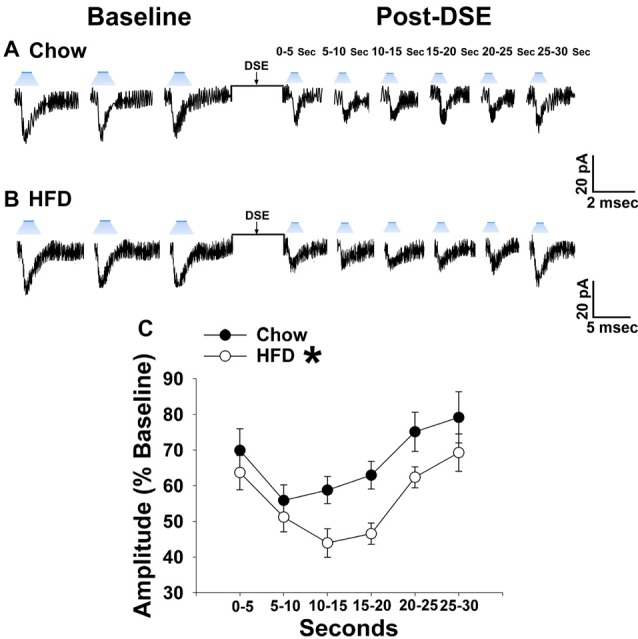
The postsynaptic depolarization-induced reduction in leEPSC amplitude seen in chow-fed males is even further reduced in HFD-fed animals. **(A)** Representative membrane current trace illustrating that the eEPSC amplitude is reduced post DSE in chow-fed animals (*n* = 22). However, this effect is significantly enhanced in animals fed a HFD (**B**; *n* = 19). A composite line graph showing how the reduction in leEPSC amplitude caused by postsynaptic depolarization is significantly augmented **(C)**. Lines represent means and vertical lines 1 SEM. *P < 0.05; rank-transformed multifactorial ANOVA/LSD.

As shown in Figures 1, 2, 5 from our guinea pig studies, long-term exposure to a “Westernized” HFD leads to insulin resistance manifested by a sexually differentiated dysregulation of energy balance and enhanced EC signaling at VMN/ARC POMC synapses. We undertook parallel studies using our mouse model to determine whether long-term exposure to a HFD could similarly induce adaptive changes in the retrograde, EC-mediated inhibition of glutamatergic transmission at VMN SF-1/ARC POMC synapses. Notably, HFD-fed NR5A1-Cre mice exhibit an obese phenotype as compared to chow-fed controls (Supplementary Figure S2). The reduction in leEPSC amplitude caused by postsynaptic depolarization is significantly accentuated during recordings in slices taken from NR5A1-Cre males fed a HFD as compared to those taken from chow-fed controls (Figures 9A–C). By contrast, we found that only a very small percentage of POMC neurons from ovariectomized, HFD-fed females exhibited leEPSCs under baseline conditions; once again effectively precluding any assessment of dietary or hormonal influences on retrograde EC-mediated signaling (Supplementary Table S3). These data demonstrate that intact male animals fed a HFD exhibit greater retrograde inhibition of glutamatergic neurotransmission at VMN SF-1/ARC POMC synapses, whereas their female counterparts do not, which further substantiates the notion that diet-induced obesity/insulin resistance is associated with a sexually differentiated enhancement of EC tone within the hypothalamic energy balance circuitry.

### Experiment #7: Does Chemostimulation of VMN SF-1 Neurons Produce Sex- and Diet- Dependent Changes in Energy Intake and Expenditure?

**Figure 10:**
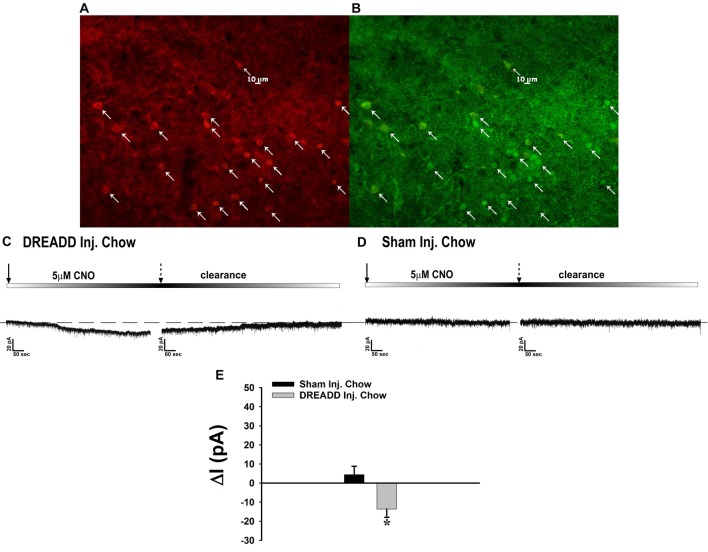
Chemostimulation of VMN SF-1 neurons in male NR5A1-cre mice reversibly stimulates POMC neurons in designer receptor exclusively activated by designer drugs (DREADD)-injected but not sham-injected animals. The color photomicrograph shows the expression of the Gq-M3-DREADD (visualized with mCherry, **A**) in SF-1 neurons (visualized with AF488, **B**). **(C)** Representative traces of membrane current showing that clozapine-N-oxide (CNO) (5 μM) induced a reversible inward current in identified POMC neurons from DREADD-injected animals (*n* = 13) that was altogether absent in cells from sham-injected animals (**D**; *n* = 4). The composite bar graph in **(E)** further illustrates the stimulatory effect of CNO in POMC neurons from DREADD-injected animals but not sham-injected animals. Bars represent means and lines 1 SEM of the CNO-induced change in membrane current. *P < 0.05; Student’s *t*-test.

**Figure 11:**
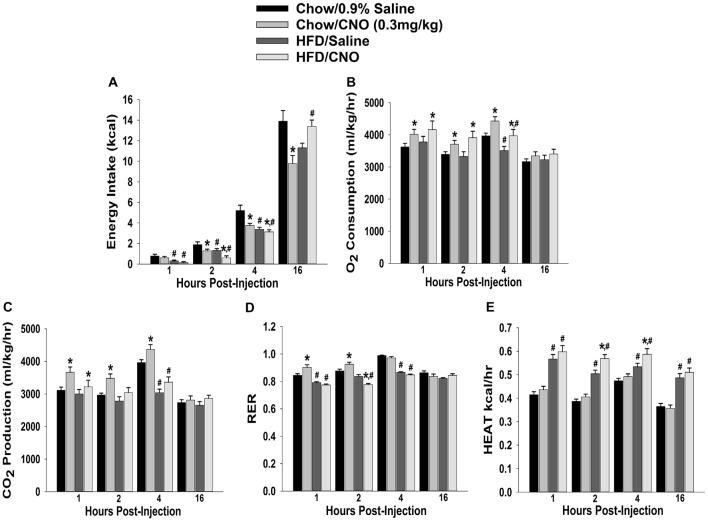
Chemostimulation of VMN SF-1 neurons in male NR5A1-cre mice reduces cumulative energy intake and increases energy expenditure in a diet-sensitive manner. CNO (0.3 mg/kg) significantly decreased cumulative energy intake **(A)**, and increased O_2_ consumption **(B)**, CO_2_ production **(C)** and RER **(D)** in chow-fed animals, all of which was significantly attenuated in HFD-fed animals. Conversely, CNO increased metabolic heat production in HFD-fed animals **(E)**. Bars represent means and lines 1 SEM of the cumulative food intake, O_2_ consumption, CO_2_ production, RER and metabolic heat production seen in chow- and HFD-fed NR5A1-Cre mice treated with either CNO (0.3 mg/kg; chow: *n* = 8; HFD: *n* = 5), or its filtered 0.9% saline vehicle (chow: *n* = 7; HFD: *n* = 5). *P < 0.05 relative to vehicle-treated animals; multifactorial ANOVA/LSD. ^#^*P* < 0.05 relative to chow-fed animals; multifactorial ANOVA/LSD.

Through our *in vitro* studies in the NR5A1-Cre mice, we have established the critical role of SF-1 neurons in providing sexually differentiated, EC- and diet-sensitive glutamatergic input onto anorexigenic POMC neurons within the hypothalamic energy balance circuitry. Therefore, it is important to ascertain the importance of these neurons in the regulation of satiety, along with the modulatory influences of sex and diet, with an *in vivo* approach. As proof of principle, we selectively activated SF-1 neurons with DREADD technology to evaluate if they in fact suppress appetite and increase energy expenditure in a sex- and diet-dependent fashion. We injected a G_q_-coupled, M3-DREADD viral vector into the dorsomedial VMN. After 2 weeks of recovery, animals were then treated subcutaneously with either CNO (0.3 kg/mL) or its vehicle saline (0.9%; 0.1 mL/kg) every day at 4 pm for the 5-day duration of the behavioral study. Immunohistofluorescence confirmed that the DREADD was indeed expressed in SF-1 neurons (Figures 10A,B), and chemostimulation of SF-1 neurons *in vitro* resulted in a pronounced and reversible inward current in POMC neurons from chow-fed, DREADD-injected but not sham-injected males (Figures 10C–E). CNO (0.3 mg/kg; s.c.) decreased cumulative energy intake in chow-fed, DREADD-injected males from two out to 16 h after its administration. In HFD-fed males; however, the CNO-induced decrease in cumulative energy intake was confined to 2–4 h post-injection, after which the animals became refractory to further CNO stimulation (Figure 11A). The CNO- and diet-induced changes in food intake were paralleled by similar changes in meal size and the rate of consumption (not shown). In terms of energy expenditure, CNO treatment increased O_2_ consumption in males from 1 h to 4 h after injection. However, the CNO-induced increase in O_2_ consumption seen in HFD-fed animals was significantly attenuated at 4 h as compared to chow-fed controls (Figure 11B). CO_2_ production was also significantly increased by CNO in chow-fed males (Figure 11C) from 1–4 h post-injection. Animals fed a HFD exhibited a significant decrease in CO_2_ production from 2–4 h post injection, and the response to CNO was limited to 1 h after administration. In addition, CNO treatment significantly increased the RER (Figure 11D) in chow-fed males out to 2 h post injection. HFD-fed animals displayed significantly decreased RER, and a CNO response that was altogether absent. On the other hand, HFD *per se* increased metabolic heat production, which was further augmented by CNO (Figure 11E). The above-described effects of CNO were not seen in chow-fed, sham-injected males (Supplementary Figures S3A–E).

**Figure 12:**
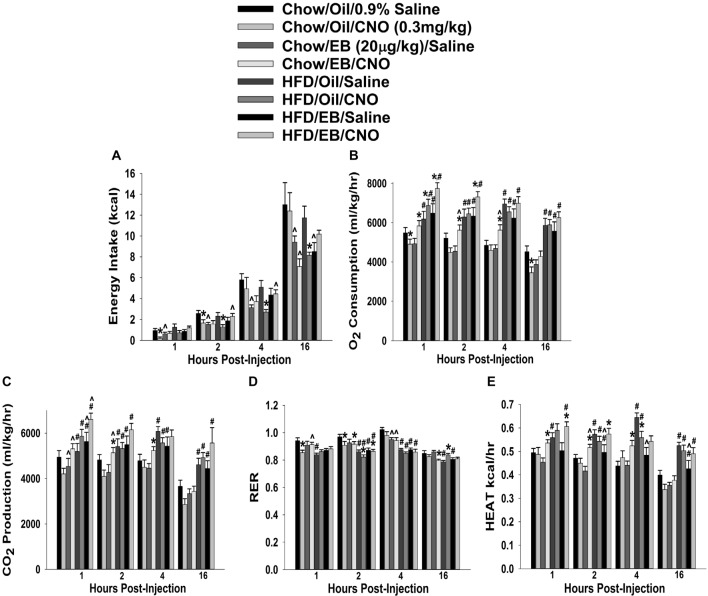
Chemostimulation of VMN SF-1 neurons in ovariectomized NR5A1-cre mice alters cumulative energy intake **(A)**, O_2_ consumption **(B)**, CO_2_ production **(C)**, RER **(D)** and metabolic heat production **(E)** in an EB- and diet-sensitive manner. CNO (0.3 mg/kg) significantly decreased energy intake in chow-fed oil-treated animals, and to a larger extent in HFD-fed animals. It also increased energy expenditure in chow- and HFD-fed, EB-treated animals. Bars represent means and lines 1 SEM of the cumulative food intake, O_2_ consumption, CO_2_ production, and RER seen in chow- and HFD-fed NR5A1-Cre mice treated with either CNO (0.3 mg/kg; oil/chow: *n* = 5; oil/HFD: *n* = 6; EB/chow: *n* = 6; EB/HFD: *n* = 5), or its filtered 0.9% saline vehicle (oil/chow: *n* = 8; oil/HFD: *n* = 6; EB/chow: *n* = 6; EB/HFD: *n* = 6). *P < 0.05 relative to saline-treated animals; multifactorial ANOVA/LSD; *n* = 5–8. ^#^*P* < 0.05 relative to chow-fed animals; multifactorial ANOVA/LSD; *n* = 5–8. ^∧^*P* < 0.05 relative to oil-treated animals; multifactorial ANOVA/LSD.

In chow-fed, sesame oil vehicle-treated ovariectomized females, CNO decreased cumulative energy intake up to 2 h post-injection (Figure 12A). EB (20 μg/kg; s.c.) also decreased intake for at least 16 h after its administration, which effectively precluded any further decrease by CNO. In HFD-fed animals, the effect of CNO was more pronounced; lasting from two to at least 16 h post-injection. EB also reduced intake at 16 h post-administration. In terms of metabolism, HFD increased all indices of energy expenditure except RER (Figures 12B–F). CNO increased O_2_ consumption in EB-treated, chow- and HFD-fed females, whereas in vehicle-treated, chow-fed CNO actually decreased it (Figure 12B). CNO also increased CO_2_ production in EB-treated, chow- but not HFD-fed females (Figure 12C). In addition, CNO decreased rather than increased RER in vehicle-treated, chow-fed animals from 1–2 h post-injection, and in EB-treated, chow-fed animals at 16 h post-administration. HFD *per se* decreased RER, which was further reduced by CNO in both vehicle- and EB-treated animals at 2 h after its injection (Figure 12D). Lastly, CNO increased metabolic heat production in EB-treated, chow- and HFD-fed animals from 1–4 and 1–2 h post-injection, respectively. Conversely, CNO decreased metabolic production in vehicle-treated, HFD-fed animals at 4 h post-administration (Figure 12E). Thus, these data indicate that chemogenetic activation of SF-1 neurons with CNO effectively decreases food intake and increases energy expenditure in a sex- and diet-dependent manner. With diet-induced obesity/insulin resistance brought on by long-term exposure to a HFD in males, the ability of these neurons to execute their functions becomes impaired, perhaps through augmented EC signaling at VMN SF-1/ARC POMC synapses. In chow-fed, vehicle-treated females, the ability of SF-1 neurons to decrease energy intake and increase energy expenditure is rather muted, again perhaps to a heightened, EC-mediated retrograde inhibition of glutamatergic input onto POMC neurons that is lost following HFD exposure. Finally, estradiol enhances the ability of SF-1 stimulation to increase energy expenditure, an effect that is retained to a large degree in HFD-animals.

## Discussion

The results from the present study demonstrate that SF-1 neurons provide a source of sexually disparate, EC-sensitive glutamatergic input onto POMC neurons that account, at least in part, for sex and dietary differences in the cannabinoid regulation for energy homeostasis. These findings are based on the following observations: (1) activation of hypothalamic CB1 receptors increases energy intake, alters meal pattern and increases energy expenditure in a sex- and diet-dependent manner; (2) chemogenetic activation of SF-1 neurons stimulates POMC neurons, suppresses food intake, alters meal pattern, and increases energy expenditure in a sex- and diet-dependent fashion; (3) EPSCs in guinea pig POMC neurons evoked by electrical stimulation of the dorsomedial VMN are subject to retrograde, EC-mediated regulation in a manner dictated by sex and dietary conditions; (4) optogenetic photostimulation elicits robust leEPSCs at VMN SF-1/ARC POMC synapses in NR5A1-cre mice that are altogether absent in NR5A1-Cre genetically modified to lack Vglut2 expression in SF-1 neurons; (5) retrograde EC-mediated inhibition of leEPSCs at VMN SF-1/ARC POMC synapses occurs to a greater extent in NR5A1-cre male mice than it does in cycling females; (6) EC tone at VMN SF-1/ARC POMC synapses is rapidly accentuated by androgens acting through membrane initiated signaling in males, an effect that is blocked by the androgen receptor antagonist flutamide and the calcium/Calmodulin inhibitor A7; (7) estradiol rapidly attenuates the EC-mediated reduction in leEPSC amplitude in ovariectomized NR5A1-Cre mice; and (8) HFD augments EC signaling at VMN SF-1/ARC POMC synapses in male guinea pigs and mice, an effect that is blocked with the Akt activator SC79.

### Testosterone Increases EC Tone at VMN SF-1/ARC POMC Synapses by Increasing Intracellular Ca^2+^ and Activating Calcium/Calmodulin-Dependent Protein Kinase

**Figure 13:**
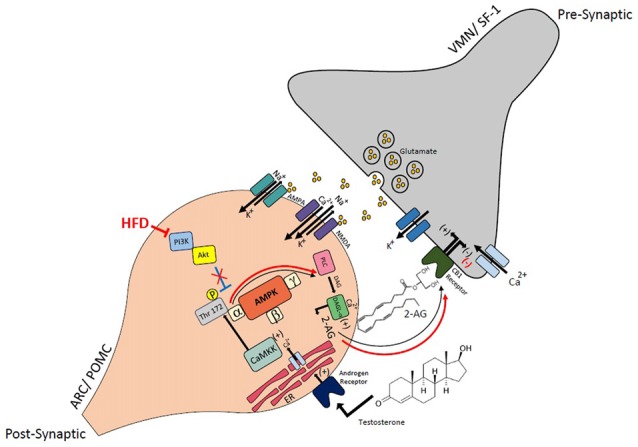
Schematic illustrating the connectivity and EC signaling occurring at VMN SF-1/ARC POMC synapses in males, and how that signaling is rapidly modulated by androgens and influenced by diet. The magnified view of the VMN SF-1/ARC POMC synapse illustrates how testosterone activates a putative membrane androgen receptor to increase intracellular calcium. This increase in intracellular calcium activates calmodulin-dependent protein kinase kinase (CaMKK), which then phosphorylates threonine 172 on the α subunit of the AMPK complex to activate the enzyme. This activation leads to an increased production of 2-arachidonylglycerol (2-AG) through diacylglycerol lipase (DAGL)α, and the 2-AG released can retrogradely activate presynaptic CB1 receptors. This inhibits calcium entry, which decreases glutamatergic input. The resultant relief from the anorexigenic signal leads to hyperphagia. AMPK activity is inhibited by PI3K/Akt signaling, and this inhibition is reduced with HFD-induced obesity/insulin resistance. This further elevates AMPK activity and subsequently EC tone at VMN SF-1/ARC POMC synapses.

We also show presently that androgens rapidly elevate EC tone at VMN SF-1/ARC POMC synapses via membrane-initiated signaling to further suppress glutamatergic input onto POMC neurons. This is accomplished by increases in intracellular Ca^2+^ and the activation of enzymes like calcium/calmodulin-dependent protein kinase and AMPK. This is consistent with what we have shown previously—namely, that testosterone increases energy intake and expenditure, which is blocked by the CB1 receptor antagonist, AM251, and increases AMPK expression in the ARC (Borgquist et al., 2015b). The membrane-initiated signaling presently observed that leads to the increased intracellular Ca^2+^ concentrations and activation of calcium/calmodulin is consistent with reports describing rapid androgenic signaling in other systems. Mitsuhashi et. al (Mitsuhashi et al., 2016) investigated the signaling pathway through which testosterone stimulates GLUT4-dependent glucose uptake into 3T3-L1 adipocytes. This study also used the membrane-impermeant TBSA that mimicked the testosterone-induced increase in glucose uptake, along with the classical androgen receptor antagonist flutamide, which had no effect. The ability of TBSA to mimic the testosterone-induced rapid increase in glucose uptake brought on by membrane-initiated signaling aligns perfectly with our results in the present study. However, the inability of flutamide to block it contrasts with what we show presently. The increase in glucose uptake was associated with the increased activity of calcium/calmodulin-dependent protein kinase I and II, as well as AMPK, which is exactly what we saw for the androgenic enhancement of EC tone at VMN SF-1/ARC POMC synapses. Other examples of rapid increases in intracellular Ca^2+^ levels elicited by membrane-initiated androgenic signaling are seen in osteoblasts and Sertoli cells, as well as skeletal muscle cells and cardiac myocytes. In all cases, the rapid signaling is triggered by the activation of a G protein-coupled androgen receptor that stimulates phospholipase C, extracellular-regulated kinase 1/2 and AMPK (Estrada et al., 2003; Vicencio et al., 2006; Loss et al., 2011; Wilson et al., 2013). Our findings show that testosterone increases EC tone by increasing intracellular Ca^2+^, which activates AMPK through calcium/calmodulin-dependent protein kinase to initiate the synthesis of 2-AG via diacylglycerol lipase (DAGL), are entirely consistent with this notion. The 2-AG produced is, in turn, released by the postsynaptic POMC neurons into the synaptic cleft, where it can engage in retrograde inhibition by activating presynaptic CB1 receptors on the terminals of the SF-1 neurons to decrease glutamate release (Figure 13).

### EC-Mediated Retrograde Inhibition of Glutamatergic Input at VMN SF-1/ARC POMC Synapses Is Sexually Differentiated and Negatively Modulated by 17β-Estradiol

Our present study shows that the degree of retrograde, EC-mediated inhibition varied depending on the stage of the estrous cycle, with a diminished retrograde inhibition during the diestrus and proestrus stages of the cycle in chow-fed NR5A1-Cre females that was significantly different from that found in estrus females or gonadally intact males. It is known that female rodents consume fewer calories during the periovulatory period straddling the proestrus and estrus stages of the estrous cycle (Asarian and Geary, 2013; Schreiber et al., 2016). This is more or less consistent with our findings that during diestrus and proestrus, when estradiol levels are rising (Asarian and Geary, 2006; Goldman et al., 2007; McLean et al., 2012), the retrograde EC-mediated inhibition of glutamate release at VMN SF-1/ARC POMC synapses is significantly attenuated. It is during this period of the estrous cycle that hypothalamic circuits are most sensitive to the actions of estradiol (Andrews et al., 1981; Gallo and Bona-Gallo, 1985). However, during estrus these same circuits become desensitized due to prolonged exposure to gradually rising levels of estradiol during the preceding stages of the cycle (Andrews et al., 1981; Gallo and Bona-Gallo, 1985). Such is the case in the present study; during estrus the degree of retrograde EC-mediated signaling at VMN SF-1/ARC POMC synapses was significantly higher than that seen during diestrus and proestrus, and comparable to that seen in gonadally intact males.

Estradiol is a potent anorexigenic hormone, and thus plays a critical role in regulating energy homeostasis in large part through the uncoupling of metabotropic G_i/o_–linked receptors, such as μ-opioid and GABA_B_ receptors, from their effector systems (Lagrange et al., 1994, 1996; Qiu et al., 2003). Hypestrogenic conditions such as those brought on by ovariectomy induce hyperphagia accompanied by increases in weight gain and adiposity, meal size, serum glucose and cholesterol (Geary and Asarian, 1999; Eckel et al., 2002; Lucas et al., 2003). In recordings from ovariectomized mice, we presently show that E_2_ significantly attenuated the postsynaptic depolarization-induced reduction in leEPSC amplitude as compared its EtOH vehicle. This indicates that estradiol is a key modulator that limits retrograde inhibition of glutamatergic input at a critical anorexigenic synapse within the hypothalamic energy circuitry.

### Diet-Induced Obesity/Insulin Resistance Profoundly Shapes the Sex Difference in the Cannabinoid-Induced Regulation of Energy Homeostasis

We have demonstrated previously that cannabinoid-induced hyperphagia and hypothermia is sexually differentiated; with males being more sensitive than their female counterparts (Wagner, 2016). This is corroborated by the findings in the present study, where the increases in energy intake, meal size and energy expenditure caused by the cannabinoid receptor agonist WIN 55,212-2 seen in chow-fed intact male guinea pigs are altogether absent in chow-fed periovulatory female guinea pigs. In fact, WIN 55,212-2 decreases rather than increases CO_2_ production and the RER in these animals. The increase in energy expenditure seen in males might be due to the sex-dependent expression of mitochondrial CB1 receptors in POMC neurons that have been reported to upregulate uncoupling protein two expression and increase mitochondrial respiration (Koch et al., 2015). By contrast, the agonist-induced hyperphagia and increase in meal size is much more pronounced in HFD-fed males, and equally dramatic increases in energy intake and meal size occur in HFD-fed periovulatory females as well. In addition, the cannabinoid-induced increase in energy expenditure observed in chow-fed males is abrogated in HFD-fed males, whereas in periovulatory females it is observed only in HFD-females. The HFD treatment paradigm produces a sexually differentiated central insulin resistance, where males but not periovulatory females exhibit a marked diminution in the insulin-induced activation of TRPC channels. This is accompanied by equally disparate changes in the ability of insulin to decrease energy intake and increase energy expenditure (Qiu et al., 2018). This differential, diet-induced insulin resistance is not associated with significant changes in adiposity, but it does cause frank dyslipidemia in both males and females (Qiu et al., 2018). This is in keeping with the fact that diet-induced insulin resistance can occur well in advance of any overt changes in body composition (Clegg et al., 2011). Moreover, adaptive changes in the excitability of neurons within the hypothalamic energy balance circuitry, as well as the density of their terminal fields, can be seen within 48 h of HFD exposure (Wei et al., 2015).

The heightened cannabinoid sensitivity that we presently show appears to occur via a different mechanism in males than it does in females. Indeed, intact male guinea pigs and NR5A1-Cre mice fed a HFD both exhibited a significantly more pronounced reduction in eEPSC amplitude at VMN SF-1/ARC POMC synapses following DSE as compared to their respective chow-fed diet controls. This is indicative of enhanced EC tone at these synapses. The diet-induced obesity/insulin resistance seen in our hands is associated with reduced PI3K/Akt signaling in the ARC (Qiu et al., 2018). Since hormones like E_2_ act through PI3K/Akt to diminish EC tone at VMN SF-1/ARC POMC synapses (Mela et al., 2016), it stands to reason that decreased ARC PI3K/Akt signaling observed with diet-induced obesity/insulin resistance in males would do just the opposite (Figure 13). Both SF-1 and POMC neurons are integral components in the hypothalamic regulation of energy homeostasis under both normophysiologic and metabolically stressed states. Indeed, the knockout of either SF-1 or any one of several components of the melanocortin system (e.g., β-endorphin, melanocortin-4 receptors) leads to hyperphagia, obesity, hyperleptinemia and hyperinsulinemia (Majdic et al., 2002; Appleyard et al., 2003; Cone, 2006). Both SF-1 and POMC neurons are modulated by peripheral hormones like leptin and insulin. They are both depolarized by leptin via PI3K-mediated activation of TRPC5 channels (Dhillon et al., 2006; Hill et al., 2008; Qiu et al., 2010). Knockout of leptin receptors in either of these neurons leads to increased adiposity and hyperleptinemia, as well as increased energy intake and decreased energy expenditure in HFD-fed animals (Balthasar et al., 2004; Dhillon et al., 2006). As shown presently and reported recently, insulin purified from guinea pig and other animal sources also depolarizes POMC neurons via a PI3K-induced activation of TRPC5 channels, and these neurons are a critical substrate in the development of sexually differentiated diet-induced insulin resistance (Qiu et al., 2014, 2018). On the other hand, Humulin and other zinc-containing insulin formulations have been shown previously to hyperpolarize POMC neurons via activation of K_ATP_ channels (Claret et al., 2007; Hill et al., 2008), an effect that can be mimicked by zinc *per se* (Qiu et al., 2014). Insulin also hyperpolarizes SF-1 neurons via PI3K-dependent K_ATP_ channel activation, and the knockout of insulin receptors in these cells protects against insulin resistance (Klöckener et al., 2011). In addition, activation of insulin receptors in the mediobasal hypothalamus promotes lipogenesis, and inhibits lipolysis and hepatic glucose production via suppression of sympathetic outflow to white adipose tissue (Scherer et al., 2011). Moreover, genetic deletion of protein-tyrosine phosphatase 1B in SF-1 neurons exacerbates diet-induced obesity in a sex-dependent manner that is associated with reduced sympathetic tone and energy expenditure in female but not male animals caused by enhanced insulin signaling in these cells (Chiappini et al., 2014). Thus, it is apparent that diet-induced obesity/insulin resistance can produce adaptive changes in the functioning of these neurons, and genetic perturbations in the functioning of these neurons can clearly impact the development of diet-induced obesity/insulin resistance.

On the other hand, we found that POMC neurons from HFD-fed periovulatory female guinea pigs and ovariectomized female NR5A1-Cre mice had substantially less excitatory input from SF-1 neurons. This is consistent with the fact that obese *ob/ob* mice lacking leptin have an increased number of excitatory inputs and a decreased number of inhibitory inputs onto NPY/AgRP neurons, as well as a reduced number of excitatory synapses impinging upon POMC neurons (Pinto et al., 2004). The strength of the excitatory input can also be diminished by fasting (Sternson et al., 2005). Thus, these adaptive changes in synaptic strength appear to be a hallmark feature of negative energy balance (in the case of fasting) as well dysregulated energy balance (in the case of obesity and insulin/leptin resistance). The question arises: what, then, accounts for the enhanced cannabinoid-induced increases in energy intake and expenditure in HFD-fed females? We have demonstrated previously that E_2_ attenuates cannabinoid-induced changes in energy balance and glutamatergic input onto POMC neurons in ovariectomized, chow-fed females (Kellert et al., 2009; Washburn et al., 2013). This is entirely consistent with our present findings, in which activation of hypothalamic cannabinoid receptors was without effect on energy intake and expenditure in chow-fed periovulatory females that are at a stage in their cycle where E_2_ predominates (Owen, 1975). The dramatic reduction in excitatory input onto POMC neurons that we presently see in HFD-fed effectively removes the primary neuroanatomical substrate upon which E_2_ normally acts to disrupt EC signaling. However, postsynaptic and mitochondrial CB1 receptors are expressed in POMC neurons, and agonist-induced activation reportedly depolarizes these cells to increase energy intake and expenditure (Koch et al., 2015). It is certainly possible that long-term HFD exposure compromises the effectiveness with which E_2_ decouples CB1 receptors from their effector systems in POMC neurons; allowing for a more robust response following CB1 receptor activation. As such, both pre- and postsynaptic receptors may play a pivotal role in providing adaptive metabolic flexibility that occurs in response to ever-changing dietary conditions. This is clearly seen in transgenic animals in which the CB1 receptor is selectively knocked in SF-1 neurons, where chow-fed animals exhibit enhanced sympathetic tone, insulin and leptin sensitivity, as well as decreased adiposity, and HFD-fed animals display just the opposite Cardinal et al. (2014). E_2_ also increases the hypothalamic expression of ionotropic glutamate receptors (Diano et al., 1997). Thus, it is also plausible that diet-induced obesity/insulin resistance compromises the ability of E_2_ to affect this increase.

Our *in vivo* chemogenetic experiments show that activation of SF-1 neurons significantly suppressed food intake and increased energy expenditure in gonadally intact, chow-fed male NR5A1-Cre animals, effects that were appreciably diminished in HFD-fed animals. These *in vivo* chemogenetic findings are largely in keeping with our *in vitro* optogenetic studies, in which animals fed a HFD exhibited augmented EC signaling at VMN SF-1/ARC POMC synapses to further reduce glutamate release onto POMC neurons. SF-1 excitation in ovariectomized females decreased energy intake and increased energy expenditure in an E_2_- and diet-dependent manner. Only modest decreases in energy intake caused by SF-1 neuronal activation were observed in chow-fed, vehicle-treated females; reminiscent of the profile seen in obese males. These effects became more robust following HFD exposure. By contrast, SF-1 stimulation elicited prominent increases in energy expenditure in EB-treated females fed either chow or HFD. That fact that these dynamic, diet-induced changes in energy balance can be observed upon SF-1 activation in the absence of functional glutamatergic synapses with POMC neurons strongly indicates that another neuromodulator (or alternatively a different downstream effector) is mediating these effects. Indeed, pituitary adenylate cyclase activating polypeptide (PACAP) is expressed by SF-1 neurons, and it reportedly suppresses energy intake and increases energy expenditure (Segal et al., 2005; Hawke et al., 2009). Future studies will determine whether PACAP is involved in the alterations in energy balance that we observe upon SF-1 neuronal activation in HFD-fed females. It was recently reported that DREADD activation occurs not via CNO, but rather by conversion to clozapine (Gomez et al., 2017). However, clozapine and other neuroleptics increase energy intake and weight gain (Fadel et al., 2002; Weston-Green et al., 2012). Given that, in our hands, chemogenetic activation of SF-1 neurons does just the opposite, we think it is unlikely that clozapine is activating the DREADDS, or any other element of the hypothalamic energy balance circuitry. Collectively, these data further support the notion that long-term exposure to HFD profoundly disrupts the hypothalamic energy balance circuitry, and the sex differences in the cannabinoid regulation of energy homeostasis.

In conclusion, the present study demonstrates that VMN SF-1/ARC POMC synapses represent an important anorexigenic component within the hypothalamic energy balance circuitry. Excitatory neurotransmission occurring at these synapses is inhibited by retrograde, EC-mediated signaling that is sexually differentiated, and enhanced under conditions of diet-induced obesity/insulin resistance in a sex-dependent manner. Collectively, these findings further advance our understanding of the hypothalamic control of energy homeostasis, which in turn will help us to develop rational, gender-based therapies for the treatment of obesity as well as HIV/AIDS- and cancer-related cachexia.

## Author Contributions

CF, JH and KC performed all stereotaxic and survival surgeries. CF, JH, SS and KC performed all electrophysiological recordings. CF, JH, RC and NA performed all metabolic studies. CF, JH, SS, KC and EW performed data analysis for all electrophysiology and metabolic studies, while NA and ST analyzed data for metabolic studies. CF and EW created all figures and performed all statistical analyses. CF and EW generated the manuscript, while CF, JH, RC, KC and EW edited the final manuscript. EW, CF and JH designed the experiments.

## Conflict of Interest Statement

The authors declare that the research was conducted in the absence of any commercial or financial relationships that could be construed as a potential conflict of interest.
