# Supplementary material for: Endocannabinoid Signaling at Hypothalamic Steroidogenic Factor-1/Proopiomelanocortin Synapses Is Sex- and Diet-Sensitive
Source: Front Mol Neurosci. 2018 Jun 19;11:214. doi: 10.3389/fnmol.2018.00214 (PMC6020785; doi:10.3389/fnmol.2018.00214)
Supplement: Supplementary file 1 [file Data_Sheet_1.pdf]

Supplementary Material

Endocannabinoid Signaling at Hypothalamic Steroidogenic Factor-1/Proopiomelanocortin Synapses is Sex- and Diet-Sensitive

Carolina Fabelo[1], Jennifer Hernandez[2], Rachel Chang[2], Sakara Seng[1], Natalia Alicea[3], Sharon Tian[1], Kristie Conde[1] & Edward J. Wagner[1, 2*]

*** Correspondence:**

Edward J. Wagner, PhD

ewagner@westernu.edu

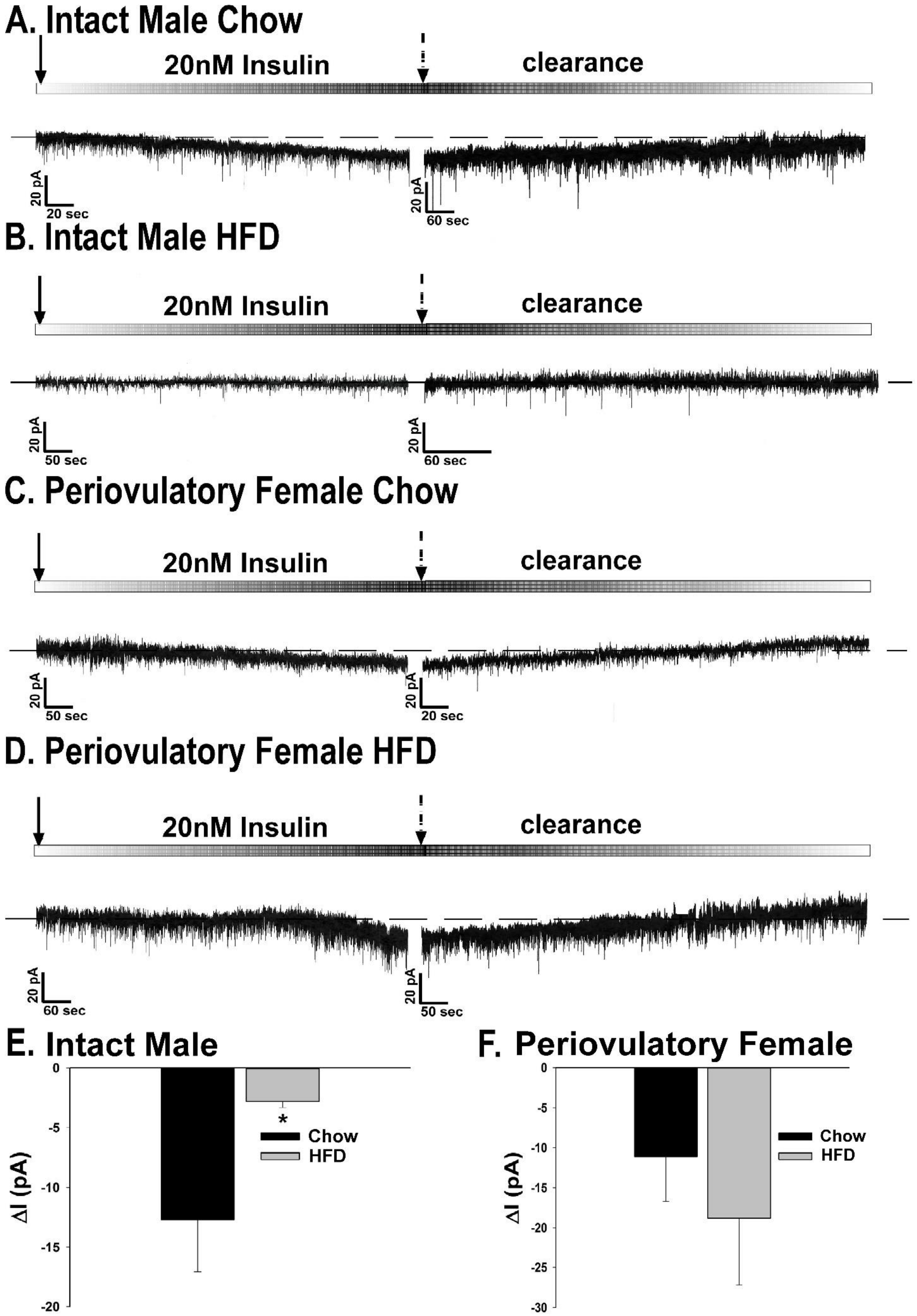
A. Intact Male Chow
20nM Insulin
clearance
20 pA
20 sec
20 pA
60 sec
B. Intact Male HFD
20nM Insulin
clearance
20 pA
50 sec
20 pA
60 sec
C. Periovulatory Female Chow
20nM Insulin
clearance
20 pA
50 sec
20 pA
20 sec
D. Periovulatory Female HFD
20nM Insulin
clearance
20 pA
60 sec
20 pA
50 sec
E. Intact Male
ΔI (pA)
0
-5
-10
-15
-20
*
Chow
HFD
F. Periovulatory Female
ΔI (pA)
0
-5
-10
-15
-20
-25
-30
Chow
HFD

Supplementary Figure 1 Chronic exposure to a HFD sex dependently attenuates the insulin-induced activation of TRPC5 channels in ARC POMC neurons. Membrane current traces of identified POMC neurons obtained from grain-based chow-fed males (**A**; n=6)), and periovulatory females (**C**; n=4) that illustrate the ability of insulin (20 nM) to cause an inward current. Membrane current traces from other identified POMC neurons taken from HFD-fed males (**B**; n=6), and periovulatory females **(D**; n=5), that illustrate how the HFD largely abolishes the insulin-induced inward current in the former but not the latter. Concurrently, thecomposite bar graphs **(**E**)** and (**F**) show the dramatic reduction of the insulin-induced inward current caused by the HFD in male but not periovulatory female animals. Bars represent means and vertical lines 1 S.E.M. of the inward current measured in POMC neurons from animals fed either a grain-based chow (open columns) or HFD (black columns). * = P < 0.05, Student's t-test.

**Male**	**% Cells with eEPSCs**	**Female**	**% Cells with eEPSCs**
**Chow**	54/72 (75%)	Chow	25/34 (74%)
**HFD**	11/16 (69%)	HFD	2/10 (20%)*

Supplementary Table 1. Proportion of POMC neurons exhibiting eEPSCs in response to electrical stimulation of the dorsomedial VMN across sex and diet. *, p < 0.05; Chi-Square test.

**Cycle Stage**	**% Responsive Cells**
**Diestrus**	16/19 (84%)
**Proestrus**	21/23 (91%)
**Estrus**	14/15 (93%)
**Metestrus**	4/12 (33%)*

Supplementary Table 2. Proportion of POMC neurons exhibiting leEPSCs across the various stages of the cycle. *, P < 0.05; Chi-Square test.

**Male**	**% Cells with eEPSCs**	**Female**	**% Cells with eEPSCs**
**Chow**	22/30 (73%)	Chow	59/76 (78%)
**HFD**	18/21 (86%)	HFD	1/10 (10%)*

Supplementary Table 3. Proportion of POMC neurons exhibiting leEPSCs in response to electrical stimulation of the dorsomedial VMN across sex and diet. *, p < 0.05; Chi-Square test.

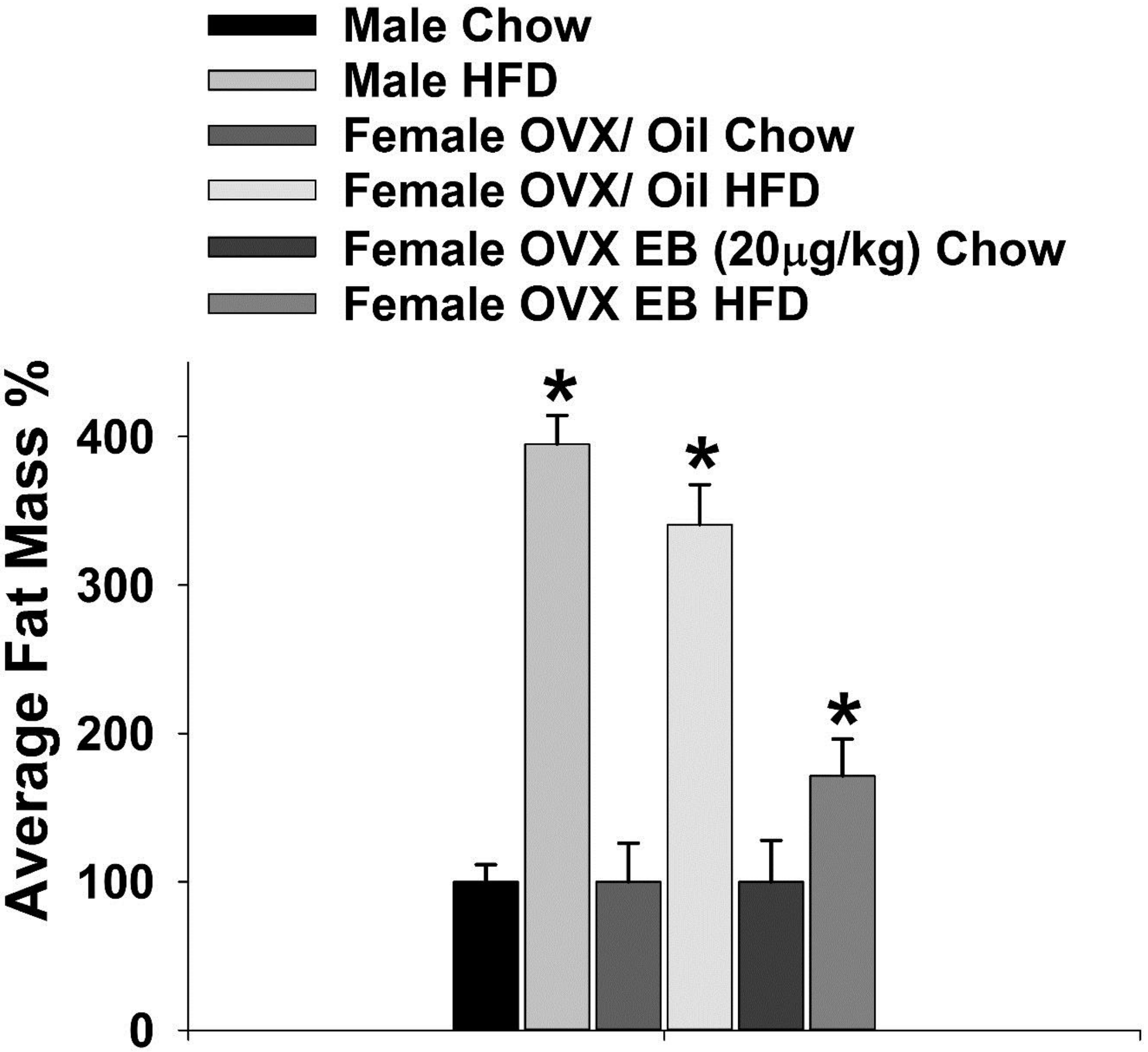

Supplementary Figure 2 HFD increases body weight and adiposity in NR5A1-Cre mice. Body weight was recorded on the day of harvest, and fat dissections were performed following harvest by which an incision was made through the umbilical region of the abdomen. Fat was collected from the dorsolumbar, inguinal, gluteal, epididymal, and perirenal regions of the abdomen and pelvic area. Bars represent means, and vertical lines 1 SEM. *P < 0.05; Student's t-test; n=5-8.

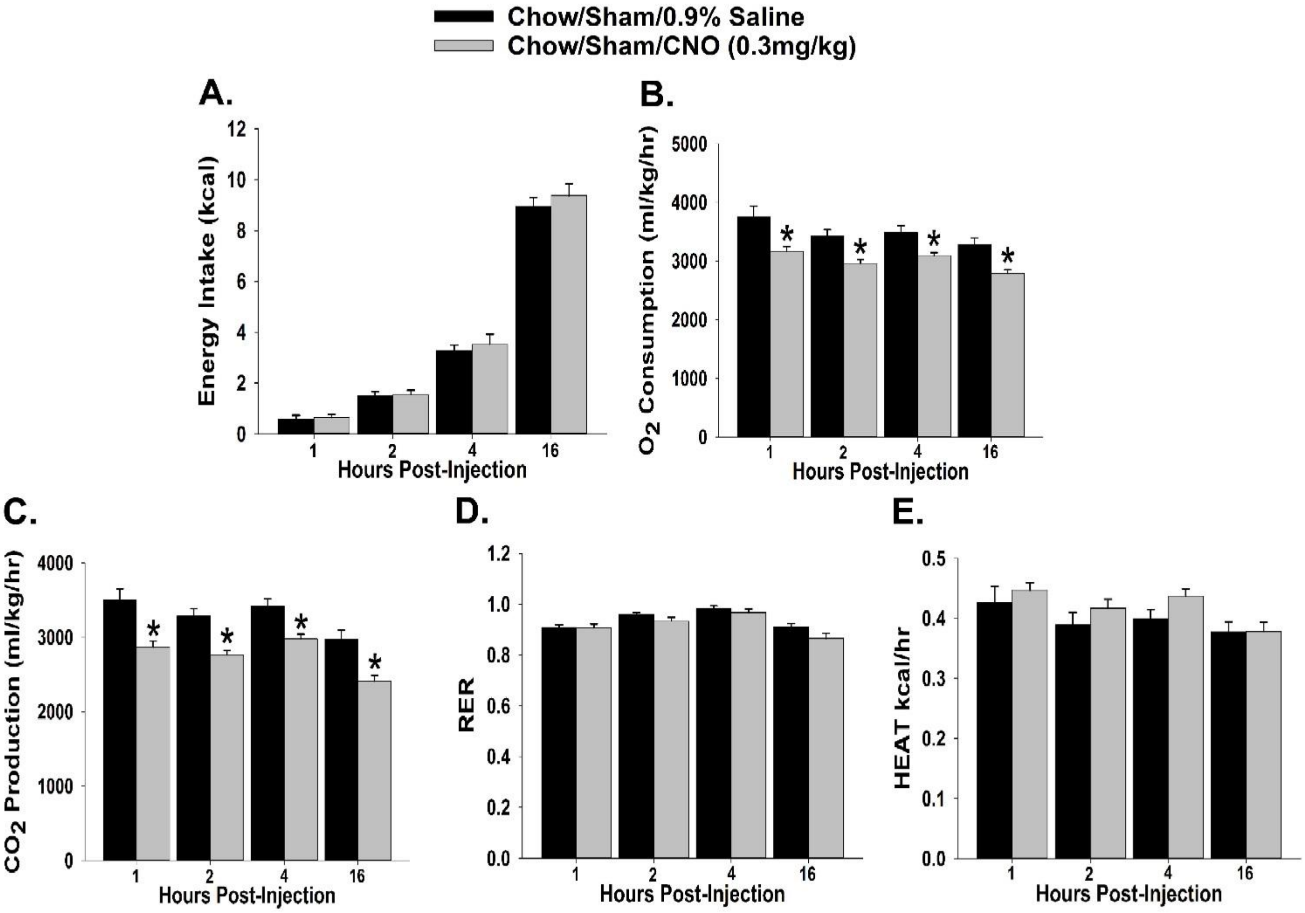

Supplementary Figure 3 CNO does not decrease energy intake or increase energy expenditure in sham-injected Nr5a1-cre male mice. CNO (0.3mg/kg) was without effect on cumulative energy intake (**A**), RER (**D**), or metabolic heat production (**E**), and actually decreased O_2 consumption (**B**) and CO_2 production (**C**). Bars represent means and lines 1 S.E.M. of the cumulative food intake, O_2 consumption, CO_2 production, RER and metabolic heat production seen in chow-fed, sham-injected NR5A1-Cre mice treated with either CNO (0.3mg/kg; n = 5), or its filtered 0.9% saline vehicle (n = 5). *, $P<0.05$; multifactorial ANOVA/LSD.
